# Impact of Data Transformation: An ECG Heartbeat Classification Approach

**DOI:** 10.3389/fdgth.2020.610956

**Published:** 2020-12-23

**Authors:** Yongbo Liang, Ahmed Hussain, Derek Abbott, Carlo Menon, Rabab Ward, Mohamed Elgendi

**Affiliations:** ^1^School of Electrical and Computer Engineering, University of British Columbia, Vancouver, BC, Canada; ^2^Faculty of Medicine, University of British Columbia, Vancouver, BC, Canada; ^3^School of Electrical and Electronic Engineering, The University of Adelaide, Adelaide, SA, Australia; ^4^Centre for Biomedical Engineering, The University of Adelaide, Adelaide, SA, Australia; ^5^Menrva Research Group, School of Mechatronic Systems Engineering and Engineering Science, Simon Fraser University, Surrey, BC, Canada; ^6^British Columbia Children's and Women's Hospital, Vancouver, BC, Canada

**Keywords:** feature mapping, feature representation, feature transformation, feature conversion, feature restructuring, data Wrangling

## Abstract

Cardiovascular diseases continue to be a significant global health threat. The electrocardiogram (ECG) signal is a physiological signal that plays a major role in preventing severe and even fatal heart diseases. The purpose of this research is to explore a simple mathematical feature transformation that could be applied to ECG signal segments in order to improve the detection accuracy of heartbeats, which could facilitate automated heart disease diagnosis. Six different mathematical transformation methods were examined and analyzed using 10s-length ECG segments, which showed that a reciprocal transformation results in consistently better classification performance for normal vs. atrial fibrillation beats and normal vs. atrial premature beats, when compared to untransformed features. The second best data transformation in terms of heartbeat detection accuracy was the cubic transformation. Results showed that applying the logarithmic transformation, which is considered the go-to data transformation, was not optimal among the six data transformations. Using the optimal data transformation, the reciprocal, can lead to a 35.6% accuracy improvement. According to the overall comparison tested by different feature engineering methods, classifiers, and different dataset sizes, performance improvement also reached 4.7%. Therefore, adding a simple data transformation step, such as the reciprocal or cubic, to the extracted features can improve current automated heartbeat classification in a timely manner.

## Introduction

Electrocardiographs (ECGs) have been a staple in medical practice for around a century. A complete heartbeat process is initiated by the sinus node—consisting of the depolarization of atriums and ventricles and the repolarization of the ventricles—in which atrial depolarization forms a P wave, ventricular depolarization forms a QRS complex wave, and the repolarization of the ventricles forms a T wave. Since its inception, ECGs have been used to diagnose physical heart abnormalities (1). When beats conform to the basic structure of a QRS complex, they are called normal beats; otherwise, they may be called arrhythmic. In an arrhythmic heartbeat—such as a beat that occurs too fast, too slow, or is irregularly timed—the morphology of the ECG waves changes accordingly.

The accurate determination of beat types can assist in the diagnosis of ECG signals. However, a more simplistic and accurate way to distinguish the heartbeats is still an unmet need. Past research has explored several ECG morphological features and many complex classifiers for achieving higher classification performance. Note that ECG morphological features (2), including RR-interval features (3) and PT-interval features (4). have been proposed, and some complex classification models, such as artificial neural networks (5), extreme learning machines (6), and deep neural networks (7) have been adopted. Although these methods can achieve slight performance improvements, the heavy computation (requiring off-line processing) limits the application of these methods. Due to the development of mobile medical technology, a greater need for robust lower computational overhead is emerging.

The rapidly increasing accessibility of mobile devices and their use in classifying different types of beats makes the investigation of different potential patterns in ECG data especially important. Any simple mathematical model can be incorporated in a mobile app to provide preliminary diagnoses of heart-related problems; this would offer patients awareness of a problem before receiving a formal diagnosis from a physician. It could also be used to promote timely self-treatment (e.g., electrolytic rebalance and breathing techniques). Recently, in 2019, Oscar et al. noted that the total number of smartphone users worldwide was projected to surpass 2.5 billion. Furthermore, the United States of America found that, as of 2017, ~64% of its population uses smartphones (8). Hence, the transformation method developed in this study was aimed for use in the growing field of mobile health. Therefore, this study focuses on whether it is possible to devise a simple feature transformation method that can improve the classification of different heartbeat events using multiple feature calculations of ECG signals without the need for complex algorithms that require high computational power to achieve similar results.

## Materials and Methods

### Hypotheses

To our knowledge, no study has investigated feature transformations to classify different heartbeat events. The main research question is: “What is the simplest mathematical transformation that can improve classification performance compared with the original features?”

### Feature Engineering

ECG signals contain a wealth of heartbeat process information, and high-quality, clear ECG signals can be used for the diagnosis and evaluation of a variety of heart diseases. However, in some cases, researchers can obtain only a small portion of the total number of ECG features. For example, ECG signals can be obtained from wearable ECG devices, but they are generally of low quality and have high interference rates, thus making it difficult to extract more accurate and effective information. In this study, to reduce the effects of noise, an 8th order 0.5–30 Hz bandpass Butterworth filter was used for the raw ECG signal (9). Meanwhile, the raw ECG signal without any filter to process (unfiltered data) was also explored in this study in order to compare the information with the filtered ECG data. Feature engineering methods in time-series biomedical signals are a common step in; for example, extraction of skewness, kurtosis, entropy, signal-noise ratio, and so on, all of which have been applied to ECG (10), photoplethysmogram (PPG) (11) and electroencephalogram (EEG) (12) signals. In this study, we adopted six feature-engineering methods to calculate ECG features based on unfiltered and filtered ECG signals (13), such as skewness (*f*_*S*_), kurtosis (*f*_*K*_), entropy (*f*_*E*_), the zero-crossing rate (*f*_*Z*_), the signal-to-noise ratio (*f*_*N*_), and relative power (*f*_*R*_). The formulas of these features are as follows:

1. **Skewness (*f***_***S***_**)**

(1) $$f_{S}=1/N\sum_{n=1}^{N}{\left[x[n]-{\hat{\mu }}_{x}/\sigma \right]}^{3}$$

where ${\hat{\mu }}_{x}\;$and σ are the empirical estimates of the mean and standard deviation of *x*, respectively, and *N* is the number of sampling points in unfiltered and filtered ECG signals.

2. **Kurtosis (*f*_***K***_**)****

(2) $$f_{S}=1/N\sum_{n=1}^{N}{\left[x[n]-{\hat{\mu }}_{x}/\sigma \right]}^{4}$$

where ${\hat{\mu }}_{x}\;$and σ are the empirical estimates of the mean and standard deviation of *x*, respectively, and *N* is the number of sampling points in unfiltered and filtered ECG signals.

3. **Entropy (*f*_***E***_**)****

(3) $$f_{E}=-\sum_{n=1}^{N}x{\left[n\right]}^{2}\log_{e}(x{\left[n\right]}^{2})$$

where *x* is the unfiltered and filtered ECG signal and N is the number of sampling points.

4. **Zero-crossing rate (*f*_***Z***_**)****

(4) $$f_{Z}=1/N\sum_{n=1}^{N}\Pi \{y<0\}$$

where *y* is the filtered ECG signal of length N and Π–the indicator function Π{*A*}–is 1 if its argument A is true, and 0 otherwise.

5. **Signal-to-noise ratio (*f*_***N***_**)****

(5) $$f_{N}=\sigma_{signal}^{2}/\sigma_{noise}^{2}$$

where σ_*signal*_ is the standard deviation of the absolute value of the unfiltered and filtered ECG signal (*y*) and σ_*noise*_ is the standard deviation of the *y* signal.

6. **Relative power (*f*_***R***_**)****

Because most of the energy of the ECG signal is concentrated within the 5–15 Hz frequency band, the ratio of the power spectral density (PSD) in this band to the PSD of the overall 1–40 Hz signal provides a measure of *f*_***R***_:


(6) $$f_{R}=\sum_{f=5}^{15}\text{PSD/}\sum_{f=1}^{40}\text{PSD}$$

where PSD is calculated using Welch's method.

### Feature Transformation

To improve features' separability, a feature transformation is applied to convert an original feature to a high dimensional space. The original input features, obtained from the previous subsection, are written as *f*. Feature transformation is a function of the input attributes φ*(f)*, defined as follows:


(7) $$\emptyset \left(f\right)=\left[\begin{array}{c} f\\ \ln\left(f\right)\\ \frac{1}{f}\\ \sqrt{f}\\ f^{2}\\ f^{3}\\ asin\left(f\right) \end{array}\right]$$

where *f* is the tested feature. This study explores whether the classification performance can be improved based only on mathematical transformations, without the use of other external features. We investigated six data transformations based on the recommendations in (14, 15).

### Database

The ECG dataset used in this study was obtained from the MIT-BIH Arrhythmia Database (16, 17), which includes ECG signals and corresponding annotated beat types. The database is made up of 30-min ECG recordings from 48 patients. For the consistency of feature calculations, we calculated the features from the 10s-long ECG signal segments; each segment was part of a separate heartbeat category. Some categories only had a small sample size, so only normal beats (Norm), atrial fibrillation (AF), atrial premature beats (APBs), and premature ventricular contractions (PVCs) were included in this study, as they had the largest sample sizes. Table 1 shows the statistics of the heartbeat segments in the MIT-BIH Arrhythmia Database, in which we can see that the number of samples in the different categories varies greatly. Specifically, the number of Norms (category 1) is four times larger than that of APBs (category 4) and two times larger than that of AFs (category 2) and PVCs (category 3). An unbalanced dataset can incorrectly represent classification performance and, because a balanced dataset was needed, a resampling technique was required. However, under-sampling and oversampling have their own flaws (18), and in order to reduce the impact of a resampling technique, two different techniques were adopted to balance the data, namely the random under-sampling technique (RUS) and the synthetic minority oversampling technique (SMOTE) (19). For the RUS process, the samples of category 1 (Norm) were resampled randomly according to the numbers in category 2 (AF), category 3 (PVC), and category 4 (APB) to classify each other. For the SMOTE process, categories 2, 3, and 4 were resampled according to the number in category 1. The RUS and SMOTE random samples were generated using MATLAB version R2019a (The MathWorks, Inc., MA, USA). Finally, RUS-balanced and SMOTE-balanced datasets were generated and used to classify the different categories, as shown in Table 2.

**Table 1:** The statistics of heartbeat segments in the MIT-BIH arrhythmia database (10).

**Index**	**Heartbeat type**	**Description**	**Number of segments**
1	Norm	Normal beat	283
2	AF	Atrial fibrillation	135
3	PVC	Premature ventricular contraction (PVC)	133
4	APB	Atrial premature beat	66
Total	(All)	-	617

*Note that the segment in this table means the 10 s length ECG segment*.

**Table 2:** The statistics of different categories in this study.

**Trial number**	**Heartbeat type**	**Number of segments (unbalanced dataset)**	**Number of segments (RUS dataset)**	**Number of segments (SMOTE dataset)**
1	Norm vs. AF	283 vs. 135	135 vs. 135	283 vs. 283
2	Norm vs. PVC	283 vs. 133	133 vs. 133	283 vs. 283
3	Norm vs. APB	283 vs. 66	66 vs. 66	283 vs. 283

*Norm, AF, PVC, and APB each represent normal beat, atrial fibrillation beat, Premature ventricular contraction, and Atrial premature beat. The unbalanced column shows that the quantity of different categories varies greatly; the balanced column shows an approximately equal number of different categories after random sampling. RUS represented random under-sampling and SMOTE represented Synthetic Minority Oversampling Technique*.

### Feature Evaluation

The original features were calculated first using six feature-engineering methods and were named *f*_*S*_, *f*_*K*_, *f*_*E*_, *f*_*Z*_, *f*_*N*_, and *f*_*R*_. New features were then developed from different dimensionalities based on these original features, including logarithmic [ln(*f*)], reciprocal (1/*f*), square-root ($\sqrt{f}$), square (*f* ^2^), cube (*f*^3^), and arcsine [asin(*f*)] calculations. To classify the different ECG categories, these ECG features were extracted and constructed based on the different feature-engineering methods and mathematical transformations. The features were evaluated by classifying the different heartbeat categories using multiple linear and non-linear classifiers.

The dataset—and each category within the dataset—was divided into a training set (70%) and a testing set (30%). In the training set, 10-fold cross-validation was adopted to validate the generalization ability of the trained classifier. In the testing phase, the performance evaluation was based on the testing set by the trained model. The F1 score was calculated as an evaluation measure as follows:


(8) F1 = 2 × Recall × Precision/(Recall + Precision)

where precision = TP/(TP + FP) and recall = TP/(TP + FN). Here, TP stands for true positives, FP stands for false positives, and FN stands for false negatives.

In order to compare the change in the classification performance, the difference measure (D-value) was adopted and calculated as follows:


(9) $$D\;value=F1_{transformed\;feature}-F1_{original\;feature}$$

If D is positive (D > 0) then the transformed feature improved the F1 accuracy, if D is zero, then transformed feature scored the same F1 score as the original feature, and if D is negative (D < 0), then the transformed feature scored less F1 score than the original feature. Certainly, the main goal of this study is to find the transformation method that is consistently achieve a D value > 0, regardless of the feature extraction method, classifier, signal quality, and sampling technique.

### Classification

**Figure 1:**
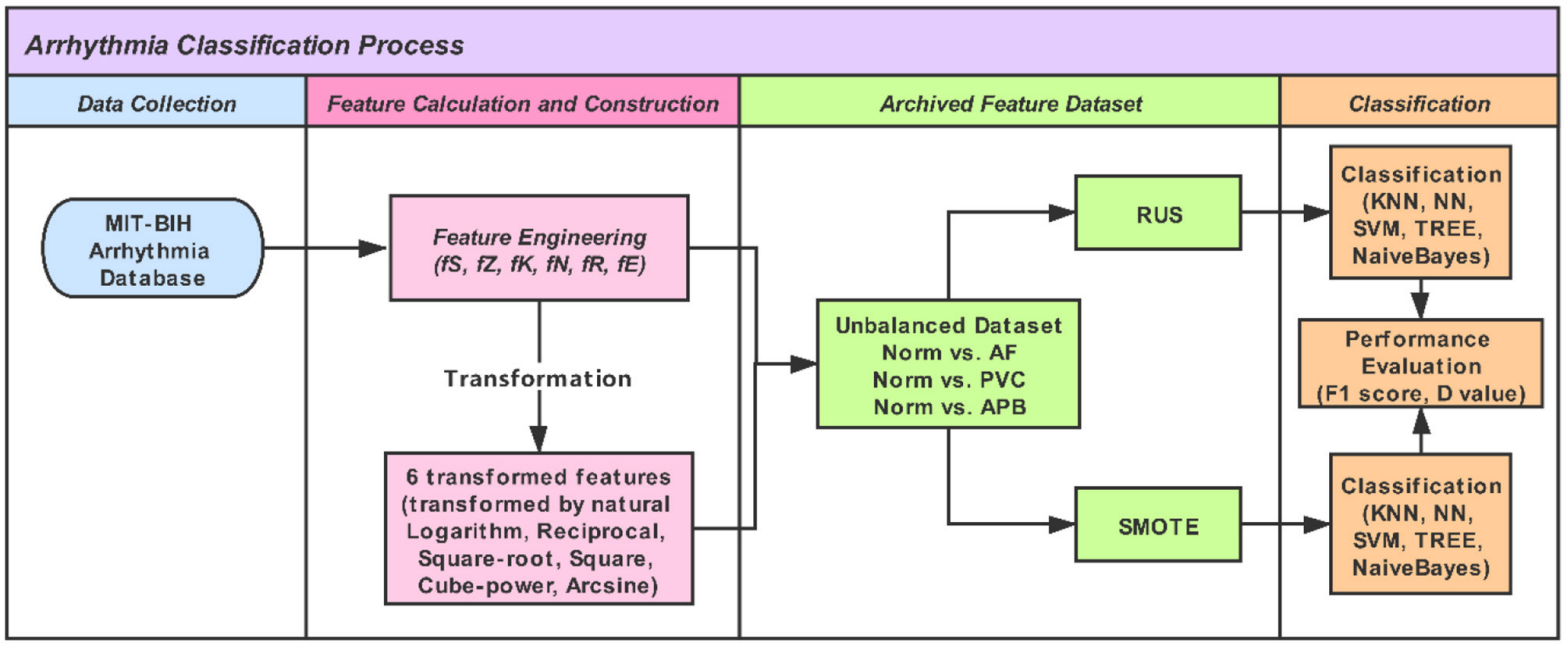
A work flowchart of this study.

We used five linear and non-linear classifiers to evaluate the different feature-engineering methods and mathematical transformations. The classifiers were the k-nearest neighbor (KNN), neural net (NN), support vector machine (SVM), decision tree (TREE), and Naïve Bayes (NB). For the KNN classifier, the number of neighbor points was set to 10. The NN classifier was a feedforward neural network with an input layer, a hidden layer with 10 neurons, and an output layer. The SVM classifier with a quadratic kernel was used. For the TREE, the split criterion of TREE was Gini's diversity index (gdi); the maximal number of decision splits was 100. For the Naïve Bayes classifer, the kernel smoother type was the Gaussian kernel, and the kernel smoothing density support was unbounded. In training the model, 10-fold cross-validation was used, which protected against overfitting by partitioning the dataset into multiple parts and estimating the accuracy of each fold. Code written in MATLAB was used to perform the feature evaluation and model training. Figure 1 shows a work flowchart of this study.

### Data Availability

The MIT-BIH Arrhythmia Database is publicly available and can be downloaded from https://www.physionet.org/physiobank/database/mitdb/.

## Results and Discussion

Each feature was used to classify different heartbeat categories, and the F1 score for each of the feature classifications was recorded and summarized in Appendix Table A. In Appendix Tables A, A.1–A.3 showed the performance of three classification trials (Norm vs. APB, Norm vs. AF, and Norm vs. PVC) that were achieved based on the unbalanced dataset. Tables A.4–A.6 showed the performance of three classification trials that were achieved based on the RUS balanced dataset. Tables A.7–A.9 showed the performance of three classification trials that were achieved based on the SMOTE balanced dataset. Each table in Appendix Table A contained the F1 score of five classifiers that were achieved by unfiltered and filtered ECG signals. The D-value in the table was calculated based on the maximum F1 score of classifiers between the original feature and the transformed features. For the original feature, the D-value was always zero as the F1 score is subtracted from itself, while the transformed feature had either a positive, negative, or zero D value. A positive value meant that the transformed feature improved the classification performance, and a negative value meant that the transformed feature was not helpful in classification. The first and last columns of Appendix Table A are the D-values. The results show that not all the mathematical transformations were consistently effective. We set the original feature as the baseline of classification with a D-value of zero. The D-value after the mathematical transformations was either positive or negative. For example, the D-value of *f*_*N*_ had the most negative value, which showed that the mathematical transformations for the signal-to-noise ratio feature were not helpful.

When we analyzed Appendix Table A, we found some interesting changes for filter processing, feature engineering, feature transformations, and different classifiers. In Table A.2, the F1 score of *f*_*S*_ is only 40% by the KNN classifier. However, the reciprocal of *f*_*S*_ ($\frac{1}{{\text{f}}_{\text{S}}})$ improves the F1 score to 75.6%. In Table A.8, the F1 score of the same solution improved from 71.6 to 91.9%. From other tables, we also found a 10% improvement of KNN after the reciprocal transformation of skewness. For filter processing, the F1 score of KNN also had a big improvement. In Table A.1, the F1 score of KNN for *f*_*Z*_, calculated by an unfiltered ECG signal, was only 18.2%, which means that the solution can't work well at all. However, after the filtering process, the F1 score of KNN reached 70%. In Table A.4, the F1 score of KNN for *f*_*Z*_ also improved from 46.5 to 73.7% after filtering. In addition, when we see the F1 score difference in classifiers, we found that KNN was not the best classifier, as it only shows a poor result compared to other classifiers. However, when we adopted some proper processing, such as filtering, transformation, and resampling methods, the performance of KNN increased from 18.2 to 95.2%, which was higher than other classifiers found in Tables A.1, A.4, A.7.

Actually, other feature engineering methods, except *f*_*Z*_, don't improve the performance of KNN. This tells us that the use of a zero-crossing rate (*f*_*Z*_) and a KNN classifier should be concerned with the noise of physiological signals, and a suitable filter should be implemented first. It is an imperative that a solution regarding the combining of optimal filter, feature engineering, feature transformation, and classifiers achieve better performance. In addition, different feature engineering methods show different characteristics. The reciprocal transformation of skewness ($\frac{1}{{\text{f}}_{\text{S}}}$) significantly improves the F1 score. However, the reciprocal transformation of other feature-engineering methods does not show improvement at all, or only shows minor improvement. For example, in Table A.2, the reciprocal transformation of skewness improved the F1 score by 35.6% for the KNN classifier, and improved the F1 score by more than 10% for other classifiers. Tables A.1, A.4, A.5, A.7–A.9 also show similar changes.

We also analyzed cases of performance reduction. In Table A.4, the D-value of ***(f***_***K***_**)**^**2**^ and the original ***f***_***K***_ was −15.1%, which demonstrates that the square transformation does not provide any help for the classification. From Table A.2, ln(*f*_*Z*_) reduces the F1 score by 4.3%, and other transformed features of ***f***_***Z***_ also had a negative D-value. As mentioned above regarding ***f***_***Z***_, the logarithm transformation of ***f***_***Z***_ wasn't helpful in improving the classification, which was not the best choice of mathematical transformation.

For different classification issues, reciprocal transformation did not improve the classification accuracy of all trials. In Table A.9, the cube transformation made the highest improvement for Norm vs. PVC classification by reaching about 9.3%. Although the reciprocal transformation also made an improvement, the improvement was only about 3.8%. In Tables A.3, A.6, cube transformation was also superior to the reciprocal transformation. According to this analysis, cube transformation had an advantage in classifying Norm and PVC compared with AF and APB.

As observed above, there was no one transformation to solve all problems. Based on Appendix Table A, we summarize the D-value as follows below:

A. **D-value = 0 (No classification accuracy improvement)**

The D-value being equal to zero means that applying feature transformation can lead to no improvement. In fact, transformed features can only provide the same result as the feature itself. As can be seen in Table A.1, when the square root was applied to the zero-crossing feature, the F1 score of ***f***_***Z***_ and $\sqrt{f_{Z}}$ were the same result (89.8%); this was achieved by the Naïve Bayes classifier. Feature transformation did not improve the classification accuracy.

For the filtered process, similar results are shown in Tables A.2, A.3, A.5, A.7; when ***f***_***S***_ was transformed to $\sqrt{f_{S}}$, the F1 score did not improve and the D-value was equal to zero. For the unfiltered process, there were also similar results, as shown in Tables A.1–A.3, A.6, A.7. The square root did not improve the performance when compared to the original feature.

B. **D-value > 0 (Classification Improvement)**

The D-value > 0 meant that the transformation improved the classification performance when compared to the original feature. We easily found the phenomenon in Table A.2 when the *f*_*Z*_ was transformed to $\frac{1}{f_{S}}$, the F1 score improved from 80.8 to 89.9%. The D-value reached up to 9.1%. In Table A.4, the D-value of (*f*_*N*_)^3^ and *f*_*N*_ reached up to 12.7% and $\frac{1}{f_{S}}$ obtained a 16% D-value in Table A.5. In Table A.8, the same transformation of $\frac{1}{f_{S}}$ improved the F1 score from 71.6 to 91.9%, which obtained a 20.3% D-value. More positive D-values can be found in Tables A.2–9.

C. **D-value < 0 (Negatively impact classification accuracy)**

In Appendix Table A, it's easy to find some negative D-values. Negative D-values mean that the transformation is terrible and is not helpful in classifying. It demonstrates that it is better not to transform for an inappropriate transformation. In Table A.4, ***(f***_***K***_**)**^**2**^ obtained a lower F1 score, from 68.1 to 52.9%, and the D-value was −15.2%. Similarly, ***(f***_***N***_**)**^**3**^ decreased the F1 score from 74.2 to 65.9% in Table A.5, and its D-value was −8.3%.

The D-value clearly shows the change of different solutions. This performance difference occurs due to the intercorrelation between the classification, feature engineering methods, and transformations. The optimal combination is valuable to explore when the available methods are limited, such as in the application with low computation and low battery.

**Figure 2:**
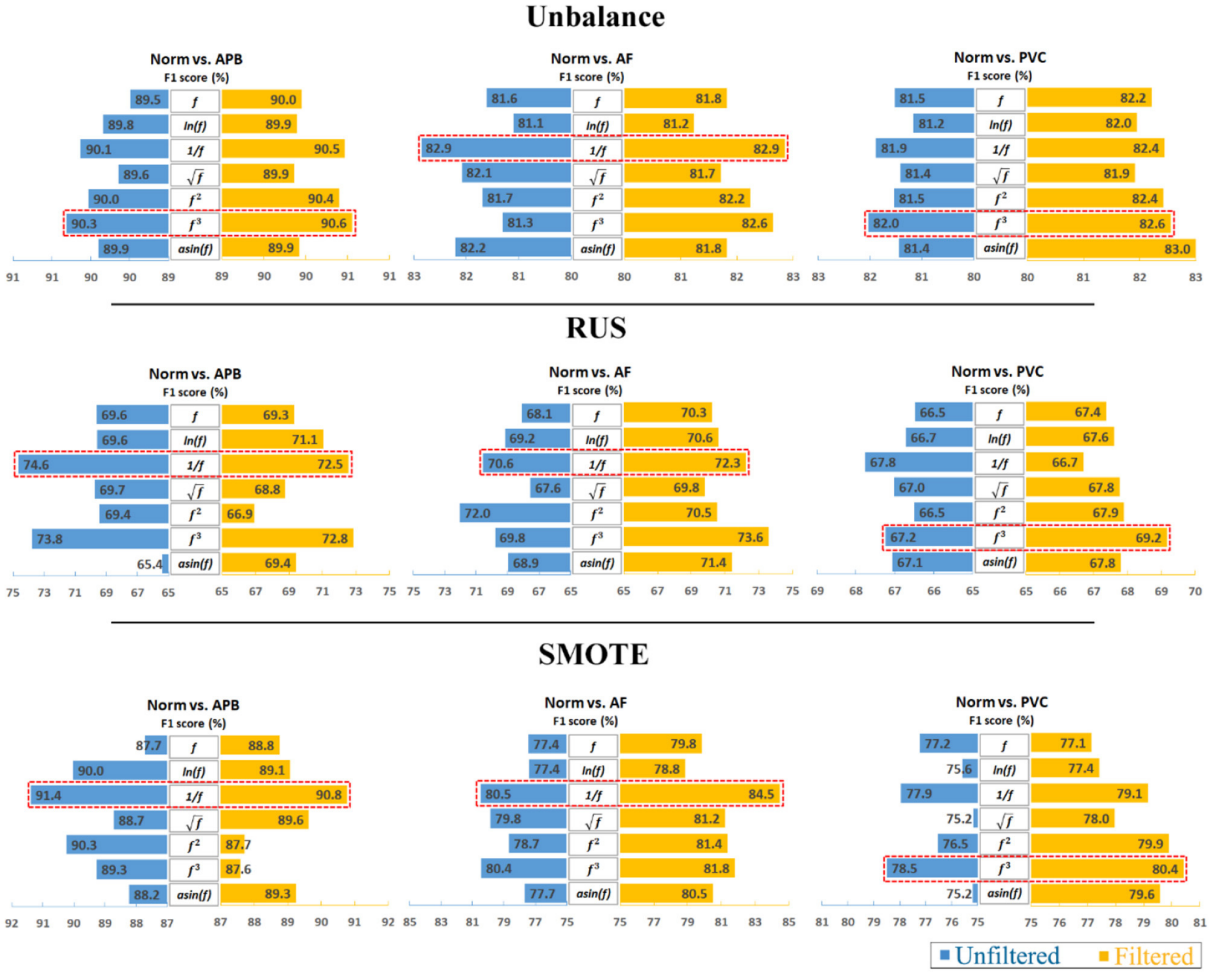
Comparison of classification performance between unfiltered data and filtered data. Note that the reciprocal is the most consistently effective data transformation.

**Figure 3:**
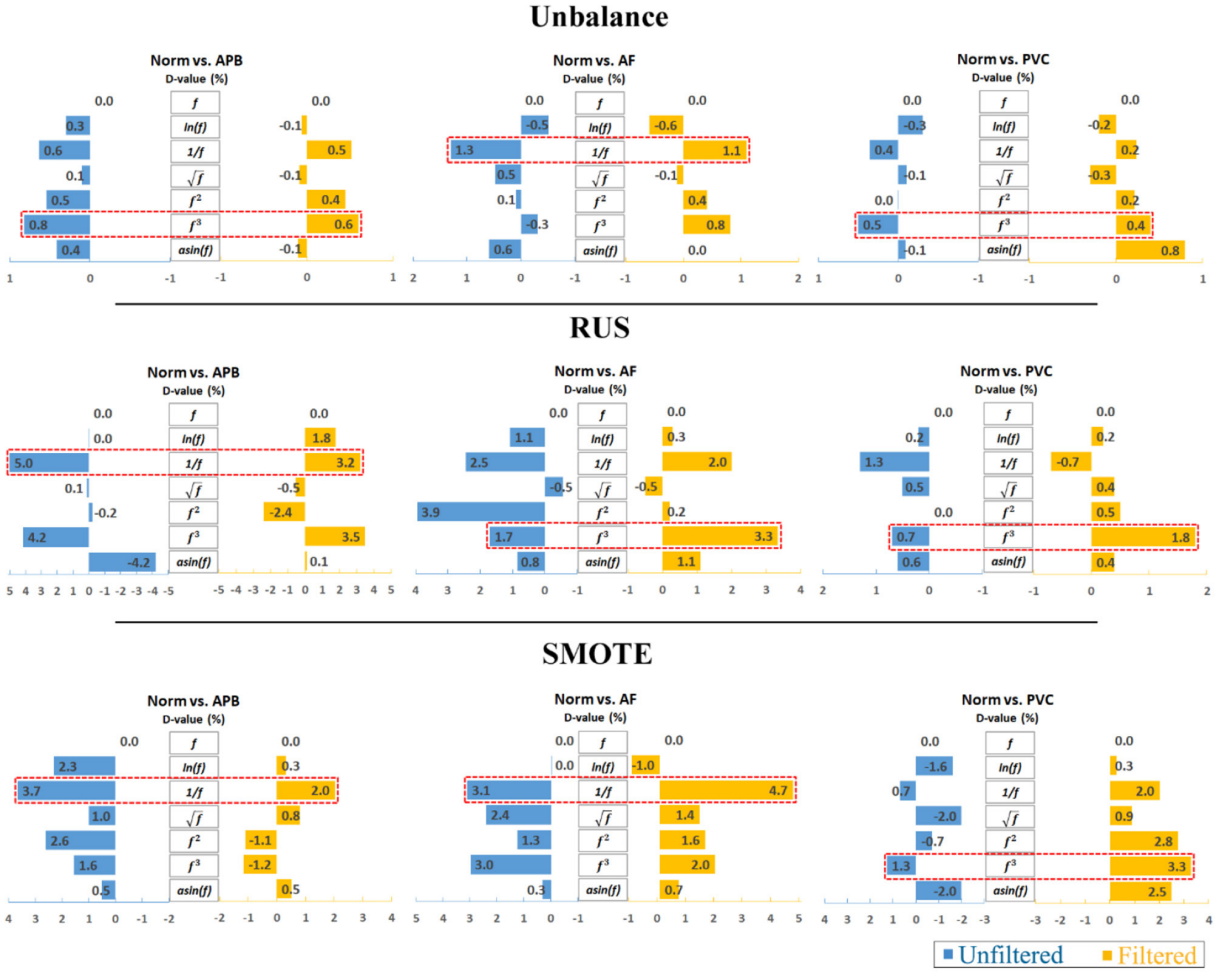
D-value comparison between the original and transformed features. Note that the reciprocal is the most consistently effective data transformation.

**Figure 4:**
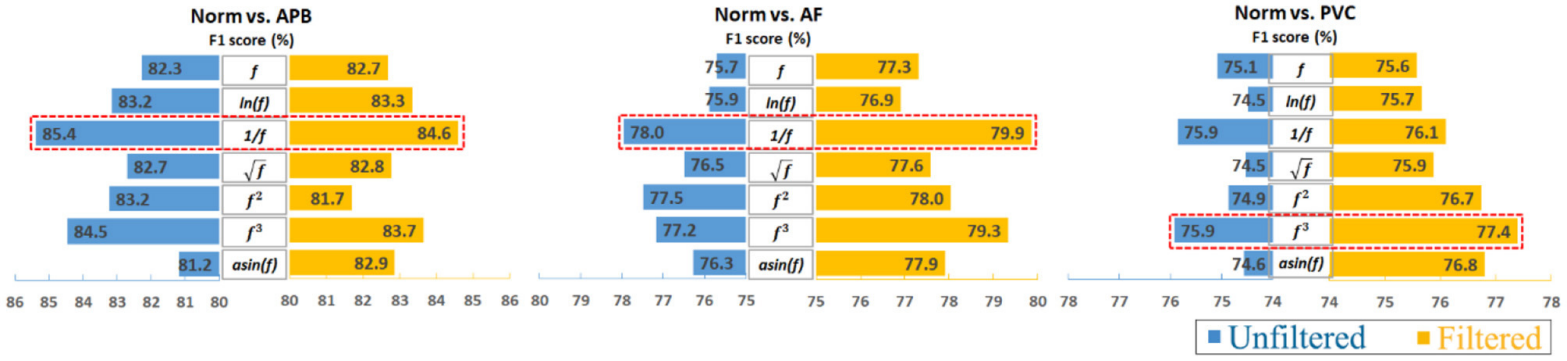
Overall impact of data transformations on classifying ECG heartbeats by averaging the unbalanced, RUS, and SMOTE results. Note that the reciprocal is the most consistently effective data transformation.

**Figure 5:**
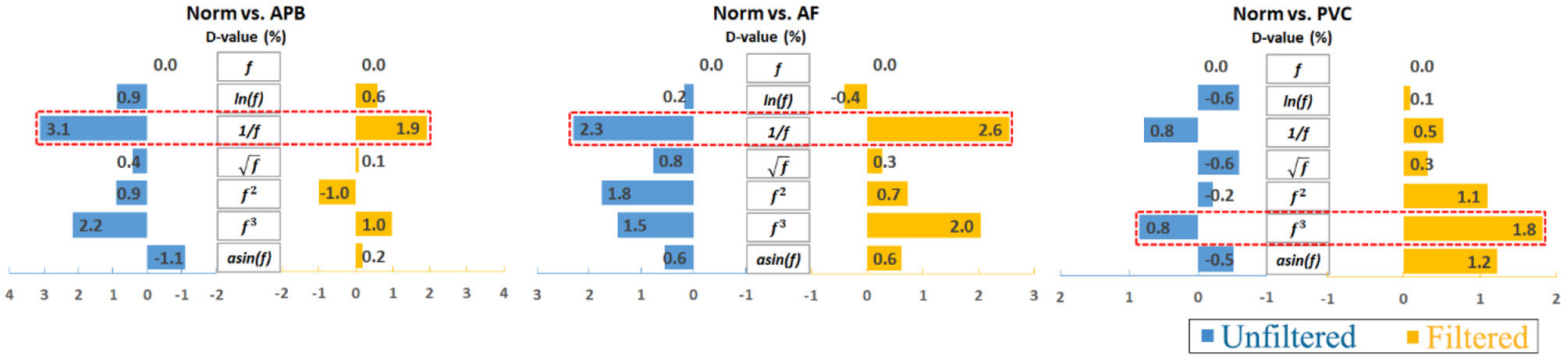
Overall D-value changes after averaging the unbalanced, RUS, and SMOTE results. Note that the reciprocal is the most consistently effective data transformation.

Furthermore, to analyze the results clearly, an average calculation for an overall analysis was conducted based on the six feature engineering methods, and the averaged results were plotted in Figure 2. Meanwhile, the corresponding D-values between original and transformed features were shown in Figure 3. From Figures 2, 3, we see that the filter improved the performance for the unbalanced, RUS, and SMOTE. Overall, the SMOTE achieved greater improvement than the unbalanced and RUS balanced datasets. In addition, most of the transformed features achieved a positive improvement compared to the original feature. In Figure 2, the red box pointed out the best transformation for each figure. According to the statistics of F1 score, $\frac{1}{f}$ and *f*^3^ made greater improvements, which were 4.7% (Norm vs. AF) and 3.3% (Norm vs. PVC). Furthermore, $\frac{1}{f}$ achieved the five best results, and *f*^3^ achieved the four best results. From these two sides, $\frac{1}{f}$ was the most stable mathematical transformation. Figure 4 shows the average F1 score of the unbalanced, RUS, and SMOTE datasets, which were a further overall calculation based on Figure 2. And Figure 5 shows the corresponding D-values between original and transformed features. The performance of Norm vs. APB, Norm vs. AF, and Norm vs. PVC were improved by 1.9, 2.6, and 1.8% by $\frac{1}{f}$, $\frac{1}{f}$, and *f*^3^, respectively.

Most of the previous research on this topic focused on the RR interval and various other morphological features. Because of the broad spectrum of varying heart diseases—as well as signal noise—ECG waveform morphologies can vary greatly. One of the most difficult issues in this field of study is the correct extraction of ECG morphological features. Although some researchers have achieved higher classification performances using methods based on hundreds of ECG morphological features (20–23), ECG signals with high levels of noise and many morphological features are usually incorrectly extracted (7, 24, 25), and this affects the robustness and effectiveness of the morphological method used. A simple feature extraction followed by optimal mathematical transformation, as this paper proposes, could be a new way to improve the detection of heart rate abnormalities.

A simple mathematical transformation, as discussed in this study, would likely not use significant processing energy or battery life and would be especially helpful in situations in which time and mobile battery life are critical. In contrast, complex calculations and classifiers, which take longer to run on even high-performance computers, would likely be less useful, as mobile devices are usually used for their simplicity and quickness, both of which the algorithm in this study promotes. However, mobile processing is evolving rapidly, so it may be possible to incorporate more complex methods into mobile devices in the near future. It is therefore important to continue improving the accuracy and reproducibility of the transformed features so they can compete with more complex methods while having the advantage of speed and simplicity.

Our findings are significant as they show the impact of mathematical transformations on extracted features and the overall accuracy in detecting abnormal heartbeats. Calculating the optimal feature (such as skewness in our study) and the optimal mathematical feature transformation is easily programmable into simple-to-use heart activity detection devices, unlike their more complex counterparts. As mentioned earlier, considering battery life, processing power, and the urgent need for an algorithm that will quickly classify heartbeats, whether for personal or medical purposes, the reciprocal feature-engineering method will perform as desired, especially considering the stable improvement in classification performance of the transformed feature.

It is also important to note that AF, PVC, and APB are some of the most prevalent arrhythmic conditions, and this algorithm will definitely be useful in the medical and self-care industry. With the development of digital health technology, wearable ECG devices are proliferating rapidly. The reciprocal transformation method used in this study is useful in this context for promoting the real-time detection of heart activity by reducing complexity and improving accuracy.

The findings of this study do not mean that we always have to use reciprocal transformation to improve classification accuracy. However, it shows the importance of feature transformation. Meanwhile, it also has some disadvantages and limitations. First, the sample size is small in this study, and more clinical data is needed to explore these new findings. Our study focused on ECG segments that are 10s in length, which only contain three types of abnormal heartbeats. Future research will explore other types of abnormalities. Second, the dataset is not balanced, which required the use of methods to upsample and downsample the dataset. Third, reciprocal transformation is applicable in solving the AF and APB detection, but it is not helpful in recognizing the PVC category. In other ways, the cube transformation is more favorable in solving the PVC category recognition. It is important to note that this is an interrelated optimization problem. In addition, given the data we have, we found that the reciprocal improves feature sensitivity and separability, which is beneficial to the classification. However, only AF, APB, and PVC were studied. More abnormal heartbeat events should be explored and validated in future work. Meanwhile, for upcoming studies, regardless of the application, we recommend testing the reciprocal as a feature transformation step and examining different classifiers for better classification results.

## Conclusion

We tested the hypothesis that a simple mathematical transformation can lead to better heartbeat classification, which could improve studies that rely on transformed features as biomarkers. Six mathematical transformations were evaluated for heart activity classification performance, and we found that some processing steps for original *f*_*Z*_ made the F1 score improve from 18.2 to 95.2%. Meanwhile, we also found that reciprocal $\frac{1}{f}$ improved the overall (tested over different feature engineering methods and different dataset sizes) classification accuracy to 4.7%. The main finding was that the application of a reciprocal transformation to features extracted from the ECG signals improved heartbeat classification consistently. The proposed extra mathematical step is therefore useful for big data analytics and can be easily incorporated into mobile and portable health applications.

## Data Availability Statement

The original contributions presented in the study are included in the article/Supplementary Materials, further inquiries can be directed to the corresponding author/s.

## Author Contributions

YL, AH, and ME processed the signals and drafted the manuscript. DA, CM, and RW advised and supervised the project. ME designed the experiment and led this investigation. All authors contributed to the article and approved the submitted version.

## Conflict of Interest

The authors declare that the research was conducted in the absence of any commercial or financial relationships that could be construed as a potential conflict of interest.
