# Supplementary material for: Impact of Data Transformation: An ECG Heartbeat Classification Approach
Source: Front Digit Health. 2020 Dec 23;2:610956. doi: 10.3389/fdgth.2020.610956 (PMC8521829; doi:10.3389/fdgth.2020.610956)
Supplement: Supplementary file 1 [file Data_Sheet_1.docx]

**Appendix Table A**

Table A.1 The classification of Normal beat versus APB based on unbalanced data. Unfiltered stands for the classification based on the raw ECG signal which don’t be processed by filter. Filtered stands for the classification based on the filtered ECG signal which is processed by a 0.5-30Hz 8^th^ order Butterworth bandpass filter. Max stands for the maximum F1 score of classifiers. D value stands for the difference value of maximum F1 score between original feature and transformed feature.

| **Unfiltered** | | | | | | | **Feature Transformation** | **Filtered** | | | | | | |
| --- | --- | --- | --- | --- | --- | --- | --- | --- | --- | --- | --- | --- | --- | --- |
| **D-value  (%)** | **Max  (%)** | **KNN  (%)** | **NN  (%)** | **SVM  (%)** | **TREE  (%)** | **Naïve Bayes (%)** |  | **KNN  (%)** | **NN  (%)** | **SVM  (%)** | **TREE  (%)** | **Naïve Bayes (%)** | **Max  (%)** | **D-value  (%)** |
| 0.0 | 89.8 | 42.9 | 87.9 | 74.5 | 83.4 | 89.8 | ***f_S_*** | 35.9 | 89.8 | 80.0 | 87.6 | 89.8 | 89.8 | 0.0 |
| 0.0 | 89.8 | 43.9 | 89.8 | 87.4 | 86.1 | 89.2 | **ln(*f_S_*)** | 40.0 | 89.8 | 82.6 | 86.9 | 89.8 | 89.8 | 0.0 |
| 1.7 | 91.6 | 66.7 | 90.2 | 4.7 | 91.5 | 91.6 | $\frac{\mathbf{1}}{\boldsymbol{f}_{\boldsymbol{S}}}$ | 33.3 | 92.3 | 92.3 | 87.9 | 92.3 | 92.3 | 2.5 |
| 0.0 | 89.8 | 51.2 | 89.8 | 28.3 | 88.2 | 89.8 | $\sqrt{\boldsymbol{f}_{\mathbf{S}}}$ | 42.4 | 89.8 | 83.9 | 90.0 | 89.8 | 90.0 | 0.2 |
| 0.1 | 89.9 | 57.1 | 89.9 | 73.3 | 86.3 | 89.8 | **(*f_S_*)^2^** | 38.1 | 93.2 | 66.2 | 82.7 | 89.8 | 93.2 | 3.3 |
| 4.4 | 94.3 | 66.7 | 94.3 | 83.0 | 88.9 | 89.8 | **(*f_S_*)^3^** | 43.8 | 92.1 | 81.3 | 87.7 | 89.8 | 92.1 | 2.3 |
| 0.0 | 89.8 | 42.1 | 88.6 | 26.5 | 84.7 | 89.8 | **asin(*f_S_*)** | 35.3 | 89.8 | 85.6 | 89.3 | 89.8 | 89.8 | 0.0 |
| 0.0 | 88.6 | 18.2 | 88.0 | 78.6 | 83.7 | 88.6 | ***f_Z_*** | 70.0 | 89.2 | 82.3 | 89.8 | 89.8 | 89.8 | 0.0 |
| 1.2 | 89.8 | 19.4 | 89.2 | 33.3 | 81.0 | 89.8 | **ln(*f_Z_*)** | 75.7 | 89.2 | 82.3 | 90.2 | 89.8 | 90.2 | 0.3 |
| 1.2 | 89.8 | 27.6 | 89.8 | 89.8 | 88.0 | 89.8 | $\frac{\mathbf{1}}{\boldsymbol{f}_{\boldsymbol{Z}}}$ | 73.7 | 89.2 | 81.6 | 89.7 | 89.8 | 89.8 | 0.0 |
| 1.2 | 89.8 | 20.0 | 89.8 | 80.0 | 83.9 | 89.2 | $\sqrt{\boldsymbol{f}_{\mathbf{Z}}}$ | 73.7 | 89.2 | 89.8 | 88.2 | 89.8 | 89.8 | 0.0 |
| 1.2 | 89.8 | 30.3 | 89.8 | 89.8 | 85.2 | 88.6 | **(*f_Z_*)^2^** | 68.3 | 89.7 | 85.6 | 89.5 | 89.8 | 89.8 | 0.0 |
| 0.0 | 88.6 | 18.2 | 88.6 | 15.2 | 84.0 | 88.6 | **(*f_Z_*)^3^** | 78.9 | 90.3 | 85.6 | 89.9 | 89.8 | 90.3 | 0.5 |
| 1.2 | 89.8 | 18.2 | 84.3 | 89.8 | 83.7 | 88.6 | **asin(*f_Z_*)** | 70.0 | 89.8 | 89.8 | 89.8 | 89.8 | 89.8 | 0.0 |
| 0.0 | 89.8 | 37.2 | 89.5 | 65.3 | 82.5 | 89.8 | ***f_K_*** | 20.0 | 89.8 | 77.8 | 85.5 | 89.8 | 89.8 | 0.0 |
| 0.0 | 89.8 | 38.9 | 88.5 | 88.4 | 84.9 | 89.8 | **ln(*f_K_*)** | 27.0 | 87.2 | 89.8 | 85.4 | 89.8 | 89.8 | 0.0 |
| 0.0 | 89.8 | 28.6 | 89.8 | 56.9 | 86.6 | 89.8 | $\frac{\mathbf{1}}{\boldsymbol{f}_{\boldsymbol{K}}}$ | 44.4 | 89.8 | 77.9 | 89.7 | 89.8 | 89.8 | 0.0 |
| 0.0 | 89.8 | 31.8 | 88.8 | 68.0 | 81.0 | 89.8 | $\sqrt{\boldsymbol{f}_{\mathbf{K}}}$ | 40.0 | 89.1 | 75.5 | 85.5 | 89.8 | 89.8 | 0.0 |
| -0.6 | 89.2 | 30.0 | 89.1 | 37.4 | 83.0 | 89.2 | **(*f_K_*)^2^** | 35.3 | 89.8 | 64.7 | 88.5 | 89.8 | 89.8 | 0.0 |
| -0.7 | 89.1 | 36.4 | 89.1 | 75.0 | 84.8 | 89.1 | **(*f_K_*)^3^** | 29.4 | 89.8 | 82.4 | 81.9 | 89.8 | 89.8 | 0.0 |
| 0.0 | 89.8 | 36.8 | 87.5 | 50.4 | 87.2 | 89.8 | **asin(*f_K_*)** | 24.2 | 90.3 | 77.1 | 88.2 | 89.8 | 90.3 | 0.5 |
| 0.0 | 88.6 | 18.2 | 88.6 | 83.5 | 85.5 | 86.8 | ***f_N_*** | 44.4 | 2.3 | 79.8 | 89.8 | 89.8 | 89.8 | 0.0 |
| 1.2 | 89.8 | 18.8 | 89.8 | 67.1 | 79.0 | 89.8 | **ln(*f_N_*)** | 40.0 | 89.8 | 79.5 | 84.7 | 89.8 | 89.8 | 0.0 |
| 1.2 | 89.8 | 36.4 | 89.8 | 61.5 | 87.2 | 89.8 | $\frac{\mathbf{1}}{\boldsymbol{f}_{\boldsymbol{N}}}$ | 57.1 | 89.2 | 86.4 | 90.2 | 89.8 | 90.2 | 0.3 |
| 0.0 | 88.6 | 24.2 | 82.8 | 82.1 | 86.0 | 88.6 | $\sqrt{\boldsymbol{f}_{\mathbf{N}}}$ | 45.7 | 89.2 | 35.0 | 89.8 | 89.2 | 89.8 | 0.0 |
| 2.0 | 90.6 | 11.1 | 90.6 | 79.0 | 79.0 | 87.8 | **(*f_N_*)^2^** | 42.4 | 89.8 | 87.3 | 86.4 | 89.2 | 89.8 | 0.0 |
| 1.2 | 89.9 | 44.4 | 89.9 | 2.3 | 87.6 | 89.3 | **(*f_N_*)^3^** | 52.9 | 90.7 | 76.1 | 91.3 | 89.5 | 91.3 | 1.5 |
| 1.6 | 90.2 | 14.6 | 90.2 | 16.8 | 80.5 | 87.4 | **asin(*f_N_*)** | 36.4 | 89.8 | 80.7 | 86.4 | 89.2 | 89.8 | 0.0 |
| 0.0 | 90.1 | 41.9 | 90.1 | 76.6 | 85.2 | 89.8 | ***f_R_*** | 21.6 | 89.8 | 32.7 | 84.8 | 89.8 | 89.8 | 0.0 |
| -0.3 | 89.8 | 48.8 | 88.1 | 82.6 | 84.8 | 89.8 | **ln(*f_R_*)** | 23.5 | 89.8 | 26.3 | 88.0 | 89.8 | 89.8 | 0.0 |
| -0.3 | 89.8 | 41.0 | 89.8 | 84.3 | 84.7 | 89.8 | $\frac{\mathbf{1}}{\boldsymbol{f}_{\boldsymbol{R}}}$ | 22.9 | 89.6 | 89.8 | 84.4 | 89.8 | 89.8 | 0.0 |
| -0.3 | 89.8 | 43.9 | 89.8 | 43.3 | 84.3 | 89.8 | $\sqrt{\boldsymbol{f}_{\mathbf{R}}}$ | 23.5 | 89.8 | 31.4 | 83.2 | 89.8 | 89.8 | 0.0 |
| -0.3 | 89.8 | 51.2 | 89.7 | 77.1 | 85.5 | 89.8 | **(*f_R_*)^2^** | 25.0 | 88.6 | 34.3 | 84.5 | 89.8 | 89.8 | 0.0 |
| 0.0 | 90.1 | 51.2 | 90.1 | 82.1 | 86.6 | 89.8 | **(*f_R_*)^3^** | 21.6 | 89.8 | 89.8 | 86.7 | 89.8 | 89.8 | 0.0 |
| -0.3 | 89.8 | 47.4 | 87.5 | 82.6 | 87.6 | 89.8 | **asin(*f_R_*)** | 31.3 | 88.0 | 34.3 | 88.9 | 89.8 | 89.8 | 0.0 |
| 0.0 | 89.8 | 31.3 | 89.8 | 78.8 | 84.6 | 89.8 | ***f_E_*** | 40.0 | 90.5 | 43.9 | 87.4 | 89.8 | 90.5 | 0.0 |
| 0.0 | 89.8 | 36.4 | 89.8 | 79.3 | 81.2 | 89.8 | **ln(*f_E_*)** | 51.3 | 89.8 | 76.3 | 86.1 | 89.8 | 89.8 | -0.7 |
| 0.0 | 89.8 | 24.2 | 89.2 | 53.5 | 84.9 | 89.8 | $\frac{\mathbf{1}}{\boldsymbol{f}_{\boldsymbol{E}}}$ | 57.9 | 90.8 | 80.7 | 87.1 | 89.8 | 90.8 | 0.3 |
| 0.0 | 89.8 | 36.4 | 89.8 | 73.8 | 83.3 | 89.8 | $\sqrt{\boldsymbol{f}_{\mathbf{E}}}$ | 47.1 | 89.8 | 49.2 | 88.4 | 89.8 | 89.8 | -0.7 |
| 0.9 | 90.7 | 43.2 | 90.7 | 52.1 | 82.9 | 89.8 | **(*f_E_*)^2^** | 47.4 | 89.8 | 83.2 | 82.6 | 89.8 | 89.8 | -0.7 |
| 0.0 | 89.8 | 31.3 | 89.8 | 74.0 | 85.2 | 89.8 | **(*f_E_*)^3^** | 40.0 | 89.8 | 54.1 | 85.5 | 89.8 | 89.8 | -0.7 |
| 0.0 | 89.8 | 36.4 | 89.8 | 79.5 | 81.9 | 89.8 | **asin(*f_E_*)** | 46.2 | 89.8 | 74.7 | 86.7 | 89.8 | 89.8 | -0.7 |

Table A.2 The classification of Normal beat versus AF based on unbalanced data. Unfiltered stands for the classification based on the raw ECG signal which don’t be processed by filter. Filtered stands for the classification based on the filtered ECG signal which is processed by a 0.5-30Hz 8^th^ order Butterworth bandpass filter. Max stands for the maximum F1 score of classifiers. D value stands for the difference value of maximum F1 score between original feature and transformed feature.

| **Unfiltered** | | | | | | | **Feature Transformation** | **Filtered** | | | | | | |
| --- | --- | --- | --- | --- | --- | --- | --- | --- | --- | --- | --- | --- | --- | --- |
| **D-value  (%)** | **Max  (%)** | **KNN  (%)** | **NN  (%)** | **SVM  (%)** | **TREE  (%)** | **Naïve Bayes (%)** |  | **KNN  (%)** | **NN  (%)** | **SVM  (%)** | **TREE  (%)** | **Naïve Bayes (%)** | **Max  (%)** | **D-value  (%)** |
| 0.0 | 80.8 | 41.0 | 80.2 | 80.8 | 74.1 | 80.8 | ***f_S_*** | 40.0 | 80.8 | 80.8 | 74.3 | 80.8 | 80.8 | 0.0 |
| 0.4 | 81.2 | 43.0 | 81.2 | 80.8 | 70.3 | 81.2 | **ln(*f_S_*)** | 44.4 | 80.8 | 80.8 | 74.9 | 81.6 | 81.6 | 0.8 |
| 9.5 | 90.2 | 69.0 | 80.2 | 80.8 | 85.7 | 90.2 | $\frac{\mathbf{1}}{\boldsymbol{f}_{\boldsymbol{S}}}$ | 75.6 | 80.2 | 80.2 | 86.4 | 89.9 | 89.9 | 9.1 |
| 0.0 | 80.8 | 48.1 | 73.6 | 80.8 | 74.7 | 80.2 | $\sqrt{\boldsymbol{f}_{\mathbf{S}}}$ | 40.5 | 75.8 | 80.8 | 75.7 | 80.8 | 80.8 | 0.0 |
| 0.0 | 80.8 | 49.3 | 80.8 | 80.8 | 80.7 | 80.8 | **(*f_S_*)^2^** | 45.3 | 82.4 | 80.8 | 78.0 | 80.8 | 82.4 | 1.6 |
| 0.4 | 81.2 | 45.8 | 79.4 | 80.8 | 76.5 | 81.2 | **(*f_S_*)^3^** | 56.8 | 78.4 | 79.6 | 81.6 | 80.8 | 81.6 | 0.8 |
| 1.8 | 82.6 | 54.8 | 80.8 | 80.2 | 75.6 | 82.6 | **asin(*f_S_*)** | 38.4 | 80.8 | 80.2 | 75.3 | 80.8 | 80.8 | 0.0 |
| 0.0 | 81.2 | 62.9 | 80.2 | 80.8 | 77.7 | 81.2 | ***f_Z_*** | 81.6 | 81.2 | 80.8 | 86.3 | 80.8 | 86.3 | 0.0 |
| -0.4 | 80.8 | 59.5 | 75.8 | 80.8 | 75.0 | 80.6 | **ln(*f_Z_*)** | 78.9 | 80.8 | 80.2 | 82.0 | 80.8 | 82.0 | -4.3 |
| -0.2 | 81.0 | 65.8 | 81.0 | 79.0 | 74.7 | 81.0 | $\frac{\mathbf{1}}{\boldsymbol{f}_{\boldsymbol{Z}}}$ | 77.9 | 80.8 | 80.8 | 82.9 | 80.8 | 82.9 | -3.5 |
| -0.4 | 80.8 | 56.8 | 76.4 | 80.8 | 77.2 | 80.2 | $\sqrt{\boldsymbol{f}_{\mathbf{Z}}}$ | 78.9 | 79.4 | 80.2 | 85.2 | 80.8 | 85.2 | -1.1 |
| 0.7 | 81.8 | 64.9 | 77.8 | 80.8 | 81.8 | 81.2 | **(*f_Z_*)^2^** | 81.6 | 70.3 | 80.8 | 84.9 | 80.8 | 84.9 | -1.4 |
| 0.1 | 81.2 | 61.5 | 80.6 | 80.8 | 81.2 | 79.4 | **(*f_Z_*)^3^** | 85.7 | 80.8 | 80.8 | 86.7 | 79.6 | 86.7 | 0.4 |
| 0.0 | 81.2 | 62.9 | 80.8 | 80.8 | 77.7 | 81.2 | **asin(*f_Z_*)** | 81.6 | 80.8 | 80.8 | 86.3 | 80.8 | 86.3 | 0.0 |
| 0.0 | 81.6 | 61.7 | 81.2 | 80.8 | 81.6 | 78.9 | ***f_K_*** | 40.5 | 75.8 | 80.8 | 70.6 | 81.2 | 81.2 | 0.0 |
| 0.4 | 82.0 | 56.4 | 77.6 | 80.8 | 76.1 | 82.0 | **ln(*f_K_*)** | 37.3 | 78.4 | 80.8 | 69.0 | 80.8 | 80.8 | -0.4 |
| -0.8 | 80.8 | 48.1 | 77.5 | 80.8 | 77.2 | 77.1 | $\frac{\mathbf{1}}{\boldsymbol{f}_{\boldsymbol{K}}}$ | 33.3 | 79.4 | 80.8 | 71.2 | 79.0 | 80.8 | -0.4 |
| 1.8 | 83.4 | 56.1 | 81.8 | 80.8 | 78.8 | 83.4 | $\sqrt{\boldsymbol{f}_{\mathbf{K}}}$ | 45.9 | 77.3 | 80.8 | 72.2 | 80.8 | 80.8 | -0.4 |
| -0.8 | 80.8 | 58.2 | 80.0 | 80.8 | 78.8 | 80.0 | **(*f_K_*)^2^** | 50.0 | 80.5 | 80.8 | 77.7 | 81.2 | 81.2 | 0.0 |
| -0.7 | 80.9 | 60.0 | 80.8 | 80.8 | 80.9 | 80.9 | **(*f_K_*)^3^** | 62.2 | 77.5 | 80.8 | 83.8 | 78.8 | 83.8 | 2.6 |
| 1.2 | 82.8 | 51.9 | 80.0 | 80.2 | 82.8 | 80.4 | **asin(*f_K_*)** | 40.5 | 78.1 | 80.8 | 71.0 | 80.8 | 80.8 | -0.4 |
| 0.0 | 84.2 | 49.3 | 84.2 | 80.8 | 74.8 | 83.9 | ***f_N_*** | 46.9 | 79.3 | 80.8 | 76.3 | 80.2 | 80.8 | 0.0 |
| -3.4 | 80.8 | 37.7 | 80.2 | 80.8 | 72.8 | 80.6 | **ln(*f_N_*)** | 45.0 | 73.3 | 80.8 | 73.8 | 71.8 | 80.8 | 0.0 |
| -2.2 | 82.0 | 52.1 | 80.8 | 80.8 | 76.0 | 82.0 | $\frac{\mathbf{1}}{\boldsymbol{f}_{\boldsymbol{N}}}$ | 49.4 | 80.2 | 80.8 | 75.6 | 79.8 | 80.8 | 0.0 |
| 1.0 | 85.2 | 41.6 | 82.1 | 80.8 | 74.9 | 85.2 | $\sqrt{\boldsymbol{f}_{\mathbf{N}}}$ | 43.9 | 78.6 | 80.2 | 67.9 | 81.6 | 81.6 | 0.8 |
| -0.1 | 84.1 | 41.6 | 83.7 | 79.6 | 75.9 | 84.1 | **(*f_N_*)^2^** | 44.7 | 78.8 | 79.6 | 76.0 | 81.6 | 81.6 | 0.8 |
| -3.4 | 80.8 | 44.7 | 75.3 | 80.8 | 72.4 | 80.7 | **(*f_N_*)^3^** | 50.0 | 81.4 | 80.8 | 76.4 | 80.2 | 81.4 | 0.6 |
| 0.4 | 84.6 | 54.1 | 83.0 | 80.2 | 79.8 | 84.6 | **asin(*f_N_*)** | 47.5 | 80.0 | 80.8 | 76.2 | 79.5 | 80.8 | 0.0 |
| 0.0 | 81.2 | 37.5 | 76.6 | 80.8 | 68.3 | 81.2 | ***f_R_*** | 27.0 | 81.2 | 80.2 | 70.5 | 81.2 | 81.2 | 0.0 |
| 0.0 | 81.2 | 39.5 | 81.2 | 80.8 | 75.3 | 81.2 | **ln(*f_R_*)** | 27.8 | 79.2 | 80.8 | 71.2 | 81.2 | 81.2 | 0.0 |
| -1.5 | 79.6 | 45.8 | 67.4 | 79.6 | 71.2 | 78.8 | $\frac{\mathbf{1}}{\boldsymbol{f}_{\boldsymbol{R}}}$ | 29.6 | 77.6 | 80.2 | 70.1 | 81.2 | 81.2 | 0.0 |
| 0.4 | 81.6 | 45.8 | 81.6 | 80.8 | 70.8 | 81.2 | $\sqrt{\boldsymbol{f}_{\mathbf{R}}}$ | 27.8 | 80.8 | 80.8 | 71.1 | 81.2 | 81.2 | 0.0 |
| 0.8 | 82.0 | 45.0 | 80.4 | 79.6 | 75.9 | 82.0 | **(*f_R_*)^2^** | 35.0 | 81.0 | 80.2 | 73.3 | 81.2 | 81.2 | 0.0 |
| 1.4 | 82.6 | 50.0 | 77.4 | 79.6 | 75.3 | 82.6 | **(*f_R_*)^3^** | 38.4 | 73.3 | 80.8 | 74.4 | 81.6 | 81.6 | 0.4 |
| 0.2 | 81.4 | 37.5 | 81.4 | 80.8 | 75.0 | 81.2 | **asin(*f_R_*)** | 33.3 | 80.8 | 80.8 | 69.7 | 81.2 | 81.2 | 0.0 |
| 0.0 | 80.8 | 33.8 | 80.8 | 80.8 | 73.7 | 78.7 | ***f_E_*** | 28.2 | 78.4 | 80.8 | 71.3 | 80.8 | 80.8 | 0.0 |
| 0.0 | 80.8 | 38.6 | 80.8 | 80.8 | 74.1 | 76.8 | **ln(*f_E_*)** | 38.5 | 81.2 | 80.8 | 73.3 | 80.8 | 81.2 | 0.4 |
| 2.8 | 83.5 | 56.8 | 83.5 | 80.8 | 75.9 | 79.0 | $\frac{\mathbf{1}}{\boldsymbol{f}_{\boldsymbol{E}}}$ | 29.4 | 81.6 | 80.8 | 75.3 | 80.2 | 81.6 | 0.8 |
| 0.0 | 80.8 | 38.5 | 80.6 | 80.8 | 71.4 | 74.1 | $\sqrt{\boldsymbol{f}_{\mathbf{E}}}$ | 38.4 | 80.8 | 80.8 | 75.3 | 80.8 | 80.8 | 0.0 |
| 0.0 | 80.8 | 38.5 | 69.3 | 80.2 | 73.1 | 80.8 | **(*f_E_*)^2^** | 37.3 | 82.2 | 80.8 | 75.6 | 80.8 | 82.2 | 1.5 |
| 0.4 | 81.2 | 44.7 | 80.8 | 80.8 | 75.3 | 81.2 | **(*f_E_*)^3^** | 38.0 | 79.4 | 79.6 | 72.1 | 80.8 | 80.8 | 0.0 |
| 0.0 | 80.8 | 32.5 | 75.6 | 80.8 | 74.4 | 76.8 | **asin(*f_E_*)** | 34.2 | 81.2 | 80.8 | 72.8 | 80.8 | 81.2 | 0.4 |

Table A.3 The classification of Normal beat versus PVC based on unbalanced data. Unfiltered stands for the classification based on the raw ECG signal which don’t be processed by filter. Filtered stands for the classification based on the filtered ECG signal which is processed by a 0.5-30Hz 8^th^ order Butterworth bandpass filter. Max stands for the maximum F1 score of classifiers. D value stands for the difference value of maximum F1 score between original feature and transformed feature.

| **Unfiltered** | | | | | | | **Feature Transformation** | **Filtered** | | | | | | |
| --- | --- | --- | --- | --- | --- | --- | --- | --- | --- | --- | --- | --- | --- | --- |
| **D-value  (%)** | **Max  (%)** | **KNN  (%)** | **NN  (%)** | **SVM  (%)** | **TREE  (%)** | **Naïve Bayes (%)** |  | **KNN  (%)** | **NN  (%)** | **SVM  (%)** | **TREE  (%)** | **Naïve Bayes (%)** | **Max  (%)** | **D-value  (%)** |
| 0.0 | 81.2 | 41.0 | 78.0 | 81.2 | 69.0 | 81.2 | ***f_S_*** | 35.1 | 81.2 | 81.2 | 72.4 | 81.2 | 81.2 | 0.0 |
| 0.8 | 82.0 | 42.1 | 81.2 | 81.2 | 72.5 | 82.0 | **ln(*f_S_*)** | 29.3 | 81.2 | 81.2 | 74.3 | 81.2 | 81.2 | 0.0 |
| 4.6 | 85.7 | 58.3 | 78.8 | 81.2 | 80.7 | 85.7 | $\frac{\mathbf{1}}{\boldsymbol{f}_{\boldsymbol{S}}}$ | 26.5 | 81.2 | 81.2 | 73.7 | 81.0 | 81.2 | 0.0 |
| 0.0 | 81.2 | 32.9 | 81.2 | 81.2 | 68.7 | 81.2 | $\sqrt{\boldsymbol{f}_{\mathbf{S}}}$ | 40.5 | 74.6 | 80.6 | 76.9 | 81.2 | 81.2 | 0.0 |
| 0.4 | 81.6 | 42.0 | 81.0 | 81.2 | 72.1 | 81.6 | **(*f_S_*)^2^** | 44.4 | 82.0 | 81.2 | 71.9 | 81.2 | 82.0 | 0.8 |
| 0.4 | 81.6 | 39.4 | 80.6 | 80.6 | 71.3 | 81.6 | **(*f_S_*)^3^** | 54.1 | 75.9 | 80.6 | 81.9 | 81.2 | 81.9 | 0.7 |
| 0.2 | 81.4 | 43.6 | 80.6 | 81.2 | 74.3 | 81.4 | **asin(*f_S_*)** | 48.7 | 85.3 | 80.6 | 80.7 | 84.0 | 85.3 | 4.1 |
| 0.0 | 81.2 | 59.7 | 67.5 | 81.2 | 78.9 | 80.0 | ***f_Z_*** | 87.1 | 78.2 | 81.2 | 84.7 | 80.6 | 87.1 | 0.0 |
| -1.2 | 80.0 | 59.7 | 80.0 | 80.0 | 75.1 | 80.0 | **ln(*f_Z_*)** | 85.7 | 69.7 | 81.2 | 82.2 | 79.4 | 85.7 | -1.3 |
| 0.0 | 81.2 | 60.5 | 80.0 | 80.6 | 74.4 | 81.2 | $\frac{\mathbf{1}}{\boldsymbol{f}_{\boldsymbol{Z}}}$ | 86.0 | 75.3 | 81.2 | 87.8 | 79.4 | 87.8 | 0.7 |
| 0.0 | 81.2 | 59.7 | 81.2 | 81.2 | 76.6 | 80.0 | $\sqrt{\boldsymbol{f}_{\mathbf{Z}}}$ | 84.7 | 69.4 | 79.4 | 85.7 | 79.4 | 85.7 | -1.3 |
| 0.0 | 81.2 | 61.3 | 80.4 | 81.2 | 78.9 | 80.0 | **(*f_Z_*)^2^** | 87.4 | 81.2 | 81.2 | 86.4 | 81.6 | 87.4 | 0.3 |
| 0.0 | 81.2 | 57.1 | 74.5 | 81.2 | 76.1 | 80.0 | **(*f_Z_*)^3^** | 89.4 | 81.1 | 81.2 | 87.8 | 81.6 | 89.4 | 2.4 |
| 0.0 | 81.2 | 59.7 | 80.0 | 81.2 | 78.9 | 80.0 | **asin(*f_Z_*)** | 87.1 | 79.4 | 81.2 | 84.7 | 80.6 | 87.1 | 0.0 |
| 0.0 | 81.2 | 32.5 | 81.2 | 80.6 | 77.5 | 81.2 | ***f_K_*** | 29.7 | 76.7 | 80.6 | 71.4 | 81.2 | 81.2 | 0.0 |
| 0.4 | 81.6 | 36.6 | 81.6 | 81.2 | 71.2 | 81.2 | **ln(*f_K_*)** | 40.0 | 80.2 | 81.2 | 72.6 | 81.2 | 81.2 | 0.0 |
| 0.0 | 81.2 | 38.4 | 81.2 | 81.2 | 78.2 | 81.2 | $\frac{\mathbf{1}}{\boldsymbol{f}_{\boldsymbol{K}}}$ | 46.9 | 82.2 | 81.2 | 68.6 | 81.2 | 82.2 | 1.1 |
| 0.4 | 81.6 | 22.5 | 81.6 | 80.0 | 74.2 | 81.2 | $\sqrt{\boldsymbol{f}_{\mathbf{K}}}$ | 33.8 | 78.6 | 81.2 | 70.2 | 81.2 | 81.2 | 0.0 |
| 0.0 | 81.2 | 31.9 | 81.2 | 81.2 | 75.6 | 80.6 | **(*f_K_*)^2^** | 33.8 | 78.4 | 81.2 | 73.9 | 81.2 | 81.2 | 0.0 |
| 4.4 | 85.6 | 38.8 | 85.6 | 81.2 | 78.7 | 80.6 | **(*f_K_*)^3^** | 48.1 | 77.8 | 81.2 | 74.1 | 81.2 | 81.2 | 0.0 |
| 0.0 | 81.2 | 34.8 | 81.2 | 81.2 | 75.3 | 81.2 | **asin(*f_K_*)** | 44.7 | 82.2 | 80.0 | 74.2 | 81.2 | 82.2 | 1.1 |
| 0.0 | 81.4 | 32.9 | 80.6 | 81.2 | 70.2 | 81.4 | ***f_N_*** | 36.1 | 81.2 | 81.2 | 73.9 | 80.6 | 81.2 | 0.0 |
| -0.2 | 81.2 | 30.0 | 81.2 | 81.2 | 63.9 | 81.2 | **ln(*f_N_*)** | 35.9 | 81.2 | 80.0 | 71.8 | 81.2 | 81.2 | 0.0 |
| -0.2 | 81.2 | 38.9 | 80.6 | 81.2 | 71.6 | 75.1 | $\frac{\mathbf{1}}{\boldsymbol{f}_{\boldsymbol{N}}}$ | 41.6 | 81.2 | 81.2 | 72.7 | 77.0 | 81.2 | 0.0 |
| 0.6 | 82.0 | 30.4 | 82.0 | 81.2 | 63.7 | 81.4 | $\sqrt{\boldsymbol{f}_{\mathbf{N}}}$ | 36.4 | 80.6 | 81.2 | 67.5 | 81.2 | 81.2 | 0.0 |
| -0.2 | 81.2 | 28.9 | 80.8 | 80.6 | 68.2 | 81.2 | **(*f_N_*)^2^** | 38.4 | 81.2 | 80.6 | 71.3 | 80.4 | 81.2 | 0.0 |
| -0.2 | 81.2 | 30.8 | 80.2 | 81.2 | 71.0 | 80.6 | **(*f_N_*)^3^** | 38.8 | 80.2 | 81.2 | 72.9 | 80.2 | 81.2 | 0.0 |
| -0.2 | 81.2 | 24.3 | 80.6 | 81.2 | 67.5 | 80.6 | **asin(*f_N_*)** | 30.8 | 80.6 | 81.2 | 67.1 | 80.6 | 81.2 | 0.0 |
| 0.0 | 83.2 | 42.5 | 80.0 | 81.2 | 74.1 | 83.2 | ***f_R_*** | 35.1 | 81.6 | 80.0 | 69.0 | 80.2 | 81.6 | 0.0 |
| -2.0 | 81.2 | 50.6 | 81.0 | 81.2 | 78.6 | 80.8 | **ln(*f_R_*)** | 33.8 | 81.4 | 81.2 | 70.9 | 81.2 | 81.4 | -0.2 |
| -2.2 | 81.0 | 47.4 | 81.0 | 80.6 | 76.7 | 81.0 | $\frac{\mathbf{1}}{\boldsymbol{f}_{\boldsymbol{R}}}$ | 46.8 | 80.4 | 81.2 | 76.6 | 81.2 | 81.2 | -0.4 |
| -1.6 | 81.6 | 47.8 | 81.6 | 81.2 | 69.2 | 80.4 | $\sqrt{\boldsymbol{f}_{\mathbf{R}}}$ | 38.4 | 80.4 | 81.2 | 71.1 | 80.0 | 81.2 | -0.4 |
| -0.2 | 83.0 | 53.2 | 67.8 | 81.2 | 77.2 | 83.0 | **(*f_R_*)^2^** | 42.1 | 81.8 | 81.2 | 75.4 | 80.6 | 81.8 | 0.2 |
| -2.0 | 81.2 | 51.3 | 81.2 | 80.6 | 80.4 | 81.2 | **(*f_R_*)^3^** | 48.0 | 80.6 | 80.6 | 75.7 | 80.6 | 80.6 | -1.0 |
| -0.6 | 82.6 | 45.8 | 80.0 | 80.6 | 70.4 | 82.6 | **asin(*f_R_*)** | 30.0 | 79.0 | 81.2 | 70.1 | 80.2 | 81.2 | -0.4 |
| 0.0 | 81.2 | 38.8 | 75.9 | 80.0 | 81.1 | 81.2 | ***f_E_*** | 48.0 | 77.7 | 81.2 | 70.4 | 81.2 | 81.2 | 0.0 |
| 0.0 | 81.2 | 40.6 | 81.2 | 81.2 | 80.2 | 81.2 | **ln(*f_E_*)** | 43.2 | 81.0 | 81.2 | 75.6 | 81.2 | 81.2 | 0.0 |
| 0.0 | 81.2 | 43.8 | 80.6 | 81.2 | 73.4 | 81.2 | $\frac{\mathbf{1}}{\boldsymbol{f}_{\boldsymbol{E}}}$ | 41.6 | 80.2 | 81.2 | 73.3 | 81.2 | 81.2 | 0.0 |
| 0.0 | 81.2 | 30.3 | 81.0 | 81.2 | 80.2 | 81.2 | $\sqrt{\boldsymbol{f}_{\mathbf{E}}}$ | 36.1 | 78.9 | 80.6 | 74.4 | 81.2 | 81.2 | 0.0 |
| 0.0 | 81.2 | 24.6 | 81.2 | 81.2 | 76.8 | 81.2 | **(*f_E_*)^2^** | 34.3 | 77.2 | 81.2 | 75.0 | 81.2 | 81.2 | 0.0 |
| 0.4 | 81.6 | 25.0 | 81.6 | 81.2 | 75.1 | 81.2 | **(*f_E_*)^3^** | 33.8 | 81.2 | 81.2 | 72.1 | 81.2 | 81.2 | 0.0 |
| 0.0 | 81.2 | 39.4 | 78.2 | 81.2 | 80.9 | 81.2 | **asin(*f_E_*)** | 43.2 | 81.2 | 81.2 | 74.7 | 81.2 | 81.2 | 0.0 |

Table A.4 The classification of Normal beat versus APB based on balanced data by RUS method. Unfiltered stands for the classification based on the raw ECG signal which don’t be processed by filter. Filtered stands for the classification based on the filtered ECG signal which is processed by a 0.5-30Hz 8^th^ order Butterworth bandpass filter. Max stands for the maximum F1 score of classifiers. D value stands for the difference value of maximum F1 score between original feature and transformed feature.

| **Unfiltered** | | | | | | | **Feature Transformation** | **Filtered** | | | | | | |
| --- | --- | --- | --- | --- | --- | --- | --- | --- | --- | --- | --- | --- | --- | --- |
| **D-value  (%)** | **Max  (%)** | **KNN  (%)** | **NN  (%)** | **SVM  (%)** | **TREE  (%)** | **Naïve Bayes (%)** |  | **KNN  (%)** | **NN  (%)** | **SVM  (%)** | **TREE  (%)** | **Naïve Bayes (%)** | **Max  (%)** | **D-value  (%)** |
| 0.0 | 73.7 | 73.7 | 59.5 | 61.5 | 61.1 | 71.4 | ***f_S_*** | 68.4 | 68.4 | 63.2 | 58.8 | 55.2 | 68.4 | 0.0 |
| -7.0 | 66.7 | 65.0 | 34.5 | 56.0 | 59.5 | 66.7 | **ln(*f_S_*)** | 69.8 | 64.9 | 66.7 | 66.7 | 74.4 | 74.4 | 6.0 |
| 18.6 | 92.3 | 89.5 | NaN | NaN | 92.3 | 89.5 | $\frac{\mathbf{1}}{\boldsymbol{f}_{\boldsymbol{S}}}$ | 66.7 | 38.9 | 63.0 | 74.4 | 68.1 | 74.4 | 6.0 |
| 0.7 | 74.4 | 60.0 | 74.4 | 61.5 | 59.5 | 72.7 | $\sqrt{\boldsymbol{f}_{\mathbf{S}}}$ | 73.2 | 64.9 | 37.0 | 65.0 | 70.6 | 73.2 | 4.8 |
| 0.7 | 74.4 | 66.7 | 74.4 | 64.2 | 55.6 | 73.7 | **(*f_S_*)^2^** | 72.7 | 70.0 | 41.4 | 53.3 | 48.3 | 72.7 | 4.3 |
| 12.8 | 86.5 | 71.8 | 57.9 | 58.1 | 68.6 | 86.5 | **(*f_S_*)^3^** | 63.2 | 58.8 | 58.1 | 58.8 | 58.1 | 63.2 | -5.3 |
| 0.7 | 74.4 | 71.4 | 68.2 | 18.2 | 57.1 | 74.4 | **asin(*f_S_*)** | 52.9 | 63.4 | 9.5 | 66.7 | 68.4 | 68.4 | 0.0 |
| 0.0 | 62.2 | 46.5 | 62.2 | 38.5 | 28.6 | 44.4 | ***f_Z_*** | 73.7 | 47.1 | 56.0 | 63.2 | 54.5 | 73.7 | 0.0 |
| 2.9 | 65.1 | 65.1 | 57.9 | 55.3 | 48.5 | 44.4 | **ln(*f_Z_*)** | 73.7 | 38.5 | 58.8 | 68.6 | 38.5 | 73.7 | 0.0 |
| 2.3 | 64.5 | 61.5 | 64.5 | 10.0 | 57.1 | 60.0 | $\frac{\mathbf{1}}{\boldsymbol{f}_{\boldsymbol{Z}}}$ | 74.3 | 54.1 | 25.0 | 65.0 | 38.5 | 74.3 | 0.6 |
| -1.8 | 60.4 | 53.7 | 41.4 | 60.4 | 47.1 | 36.4 | $\sqrt{\boldsymbol{f}_{\mathbf{Z}}}$ | 76.9 | 41.4 | 61.5 | 53.3 | 37.0 | 76.9 | 3.2 |
| -2.2 | 60.0 | 45.5 | 60.0 | 59.1 | 29.4 | 51.3 | **(*f_Z_*)^2^** | 78.9 | 56.4 | 66.7 | 78.0 | 54.1 | 78.9 | 5.3 |
| 1.9 | 64.2 | 54.5 | 50.0 | 64.2 | 36.4 | 62.2 | **(*f_Z_*)^3^** | 81.1 | 44.4 | 66.7 | 68.3 | 51.3 | 81.1 | 7.4 |
| -15.7 | 46.5 | 46.5 | 45.0 | 25.0 | 28.6 | 44.4 | **asin(*f_Z_*)** | 73.7 | 48.6 | 64.2 | 63.2 | 54.5 | 73.7 | 0.0 |
| 0.0 | 71.8 | 62.9 | 66.7 | 50.0 | 71.8 | 50.0 | ***f_K_*** | 57.1 | 57.1 | 68.1 | 60.0 | 22.2 | 68.1 | 0.0 |
| 2.6 | 74.4 | 70.3 | 23.1 | 55.8 | 74.4 | 54.5 | **ln(*f_K_*)** | 60.0 | 56.4 | 65.0 | 68.4 | 45.7 | 68.4 | 0.3 |
| -8.6 | 63.2 | 59.5 | 63.2 | 41.0 | 63.2 | 54.1 | $\frac{\mathbf{1}}{\boldsymbol{f}_{\boldsymbol{K}}}$ | 73.2 | 52.6 | 70.4 | 66.7 | 50.0 | 73.2 | 5.1 |
| 1.4 | 73.2 | 70.3 | 55.0 | 66.7 | 73.2 | 45.2 | $\sqrt{\boldsymbol{f}_{\mathbf{K}}}$ | 56.4 | 41.2 | 36.4 | 51.3 | 38.7 | 56.4 | -11.7 |
| -1.8 | 70.0 | 62.9 | 63.4 | 61.2 | 70.0 | 45.2 | **(*f_K_*)^2^** | 52.9 | 29.6 | 34.5 | 51.4 | 37.0 | 52.9 | -15.2 |
| -8.4 | 63.4 | 63.4 | 53.3 | 23.1 | 62.5 | 58.8 | **(*f_K_*)^3^** | 64.9 | 52.6 | 55.3 | 66.7 | 51.6 | 66.7 | -1.4 |
| 4.4 | 76.2 | 66.7 | 66.7 | 28.6 | 76.2 | 50.0 | **asin(*f_K_*)** | 65.0 | 54.5 | 73.5 | 66.7 | 45.7 | 73.5 | 5.4 |
| 0.0 | 64.2 | 54.1 | 47.6 | 64.2 | 56.4 | 60.0 | ***f_N_*** | 61.9 | 41.4 | 69.1 | 48.5 | 48.6 | 69.1 | 0.0 |
| 4.4 | 68.6 | 68.6 | 64.5 | 10.0 | 65.0 | 50.0 | **ln(*f_N_*)** | 63.4 | 42.9 | 68.0 | 57.1 | 38.7 | 68.0 | -1.1 |
| 19.6 | 83.7 | 76.5 | 76.9 | 45.8 | 83.7 | 76.5 | $\frac{\mathbf{1}}{\boldsymbol{f}_{\boldsymbol{N}}}$ | 56.4 | 32.0 | 69.0 | 64.9 | 64.5 | 69.0 | -0.1 |
| 2.5 | 66.7 | 63.2 | 54.1 | 9.5 | 66.7 | 38.5 | $\sqrt{\boldsymbol{f}_{\mathbf{N}}}$ | 53.7 | 68.1 | 65.4 | 50.0 | 45.7 | 68.1 | -1.0 |
| 3.8 | 68.0 | 55.6 | 61.9 | 9.5 | 57.8 | 68.0 | **(*f_N_*)^2^** | 65.0 | 41.2 | 69.1 | 62.9 | 54.1 | 69.1 | 0.0 |
| 13.1 | 77.3 | 57.1 | 58.8 | 55.6 | 74.4 | 77.3 | **(*f_N_*)^3^** | 66.7 | 8.0 | 67.9 | 73.7 | 81.8 | 81.8 | 12.7 |
| -18.0 | 46.2 | 46.2 | 45.7 | 38.5 | 45.7 | 37.0 | **asin(*f_N_*)** | 56.4 | 15.4 | 67.0 | 51.4 | 44.4 | 67.0 | -2.1 |
| 0.0 | 77.8 | 48.6 | 66.7 | 65.5 | 51.6 | 77.8 | ***f_R_*** | 55.6 | 59.6 | 40.0 | 60.5 | 57.8 | 60.5 | 0.0 |
| -3.5 | 74.3 | 70.0 | 59.1 | 8.3 | 68.6 | 74.3 | **ln(*f_R_*)** | 40.0 | 64.9 | 42.9 | 59.1 | 60.5 | 64.9 | 4.4 |
| 1.2 | 78.9 | 68.1 | 70.6 | 68.2 | 53.3 | 78.9 | $\frac{\mathbf{1}}{\boldsymbol{f}_{\boldsymbol{R}}}$ | 47.1 | 63.4 | 57.1 | 55.8 | 60.5 | 63.4 | 3.0 |
| -3.5 | 74.3 | 60.0 | 55.0 | 68.3 | 58.1 | 74.3 | $\sqrt{\boldsymbol{f}_{\mathbf{R}}}$ | 45.2 | 46.2 | 55.3 | 60.9 | 59.1 | 60.9 | 0.4 |
| -3.5 | 74.3 | 61.5 | 63.2 | 65.3 | 48.3 | 74.3 | **(*f_R_*)^2^** | 45.7 | 55.8 | 56.5 | 51.3 | 56.5 | 56.5 | -3.9 |
| -0.9 | 76.9 | 52.4 | 70.6 | 16.7 | 58.8 | 76.9 | **(*f_R_*)^3^** | 47.1 | 60.5 | 19.0 | 65.2 | 63.8 | 65.2 | 4.8 |
| 0.0 | 77.8 | 43.9 | 62.9 | 68.0 | 56.3 | 77.8 | **asin(*f_R_*)** | 54.1 | 51.4 | 42.9 | 52.6 | 57.8 | 57.8 | -2.7 |
| 0.0 | 68.1 | 58.5 | 68.1 | 9.1 | 63.4 | 64.9 | ***f_E_*** | 68.4 | 69.8 | 55.8 | 76.2 | 68.4 | 76.2 | 0.0 |
| 0.3 | 68.4 | 51.3 | 46.7 | 25.0 | 65.1 | 68.4 | **ln(*f_E_*)** | 75.7 | 76.9 | 37.5 | 76.2 | 68.4 | 76.9 | 0.7 |
| -2.9 | 65.2 | 52.9 | 65.2 | 10.0 | 65.0 | 65.1 | $\frac{\mathbf{1}}{\boldsymbol{f}_{\boldsymbol{E}}}$ | 72.2 | 75.0 | 66.7 | 68.2 | 81.0 | 81.0 | 4.8 |
| 1.5 | 69.6 | 52.6 | 69.6 | 52.4 | 65.0 | 64.9 | $\sqrt{\boldsymbol{f}_{\mathbf{E}}}$ | 68.6 | 75.0 | 37.5 | 77.3 | 68.4 | 77.3 | 1.1 |
| 1.7 | 69.8 | 48.6 | 69.8 | 34.5 | 55.0 | 62.5 | **(*f_E_*)^2^** | 64.9 | 41.2 | 37.5 | 71.4 | 68.4 | 71.4 | -4.8 |
| 6.3 | 74.4 | 64.9 | 74.4 | 66.7 | 60.0 | 53.3 | **(*f_E_*)^3^** | 58.8 | 79.1 | 20.7 | 66.7 | 52.9 | 79.1 | 2.9 |
| 3.3 | 71.4 | 51.3 | 71.4 | NaN | 65.1 | 68.4 | **asin(*f_E_*)** | 75.7 | 71.8 | 37.5 | 76.2 | 68.4 | 76.2 | 0.0 |

Table A.5 The classification of Normal beat versus AF based on balanced data by RUS method. Unfiltered stands for the classification based on the raw ECG signal which don’t be processed by filter. Filtered stands for the classification based on the filtered ECG signal which is processed by a 0.5-30Hz 8^th^ order Butterworth bandpass filter. Max stands for the maximum F1 score of classifiers. D value stands for the difference value of maximum F1 score between original feature and transformed feature.

| **Unfiltered** | | | | | | | **Feature Transformation** | **Filtered** | | | | | | |
| --- | --- | --- | --- | --- | --- | --- | --- | --- | --- | --- | --- | --- | --- | --- |
| **D-value  (%)** | **Max  (%)** | **KNN  (%)** | **NN  (%)** | **SVM  (%)** | **TREE  (%)** | **Naïve Bayes (%)** |  | **KNN  (%)** | **NN  (%)** | **SVM  (%)** | **TREE  (%)** | **Naïve Bayes (%)** | **Max  (%)** | **D-value  (%)** |
| 0.0 | 64.9 | 53.3 | 63.9 | 64.4 | 59.0 | 64.9 | ***f_S_*** | 57.8 | 65.2 | 66.7 | 51.3 | 63.4 | 66.7 | 0.0 |
| 1.8 | 66.7 | 64.2 | 57.1 | 66.7 | 60.5 | 50.8 | **ln(*f_S_*)** | 59.7 | 59.5 | 64.4 | 47.4 | 69.1 | 69.1 | 2.5 |
| 15.6 | 80.5 | 77.6 | 17.8 | 66.7 | 69.3 | 80.5 | $\frac{\mathbf{1}}{\boldsymbol{f}_{\boldsymbol{S}}}$ | 75.0 | 68.8 | 65.5 | 79.5 | 82.7 | 82.7 | 16.0 |
| 1.8 | 66.7 | 63.5 | 65.9 | 66.7 | 59.0 | 64.9 | $\sqrt{\boldsymbol{f}_{\mathbf{S}}}$ | 56.4 | 63.6 | 66.7 | 55.8 | 65.1 | 66.7 | 0.0 |
| 6.6 | 71.4 | 70.0 | 71.4 | 66.7 | 70.7 | 64.9 | **(*f_S_*)^2^** | 64.0 | 65.9 | 66.7 | 66.7 | 67.5 | 67.5 | 0.8 |
| 1.8 | 66.7 | 56.0 | 66.7 | 65.5 | 61.0 | 63.3 | **(*f_S_*)^3^** | 62.2 | 70.9 | 66.7 | 65.1 | 72.3 | 72.3 | 5.6 |
| 2.7 | 67.6 | 60.0 | 63.6 | 66.7 | 55.7 | 67.6 | **asin(*f_S_*)** | 56.0 | 62.2 | 66.7 | 62.5 | 61.5 | 66.7 | 0.0 |
| 0.0 | 65.9 | 56.3 | 44.8 | 64.4 | 65.9 | 57.1 | ***f_Z_*** | 79.5 | 63.4 | 66.7 | 63.3 | 54.5 | 79.5 | 0.0 |
| 0.8 | 66.7 | 65.0 | 53.7 | 66.7 | 64.3 | 55.1 | **ln(*f_Z_*)** | 80.5 | 42.3 | 64.4 | 65.9 | 52.6 | 80.5 | 1.0 |
| -2.4 | 63.4 | 52.8 | 62.2 | 63.2 | 61.4 | 63.4 | $\frac{\mathbf{1}}{\boldsymbol{f}_{\boldsymbol{Z}}}$ | 79.5 | 67.9 | 66.7 | 55.6 | 45.6 | 79.5 | 0.0 |
| 0.8 | 66.7 | 53.8 | 66.7 | 64.4 | 54.8 | 57.1 | $\sqrt{\boldsymbol{f}_{\mathbf{Z}}}$ | 80.5 | 52.1 | 66.7 | 67.5 | 52.1 | 80.5 | 1.0 |
| 6.7 | 72.5 | 63.0 | 50.7 | 66.7 | 72.5 | 58.0 | **(*f_Z_*)^2^** | 77.9 | 31.7 | 66.7 | 68.2 | 56.3 | 77.9 | -1.6 |
| 2.3 | 68.1 | 61.3 | 60.0 | 66.7 | 68.1 | 53.7 | **(*f_Z_*)^3^** | 82.5 | 54.5 | 66.7 | 68.9 | 59.0 | 82.5 | 3.0 |
| 0.0 | 65.9 | 56.3 | 55.6 | 64.4 | 65.9 | 57.1 | **asin(*f_Z_*)** | 79.5 | 52.6 | 65.5 | 63.3 | 54.5 | 79.5 | 0.0 |
| 0.0 | 67.5 | 67.5 | 56.8 | 66.7 | 62.5 | 59.5 | ***f_K_*** | 53.3 | 56.8 | 66.7 | 60.0 | 48.6 | 66.7 | 0.0 |
| 4.6 | 72.1 | 72.1 | 56.0 | 66.7 | 67.6 | 56.4 | **ln(*f_K_*)** | 51.9 | 46.2 | 66.7 | 55.2 | 48.1 | 66.7 | 0.0 |
| 0.0 | 67.5 | 57.5 | 57.5 | 66.7 | 67.5 | 59.0 | $\frac{\mathbf{1}}{\boldsymbol{f}_{\boldsymbol{K}}}$ | 47.4 | 12.8 | 66.7 | 58.8 | 50.6 | 66.7 | 0.0 |
| -0.8 | 66.7 | 55.3 | 63.6 | 66.7 | 66.7 | 56.8 | $\sqrt{\boldsymbol{f}_{\mathbf{K}}}$ | 65.1 | 56.1 | 65.5 | 57.8 | 41.7 | 65.5 | -1.1 |
| 8.1 | 75.6 | 64.1 | 72.9 | 65.5 | 68.2 | 75.6 | **(*f_K_*)^2^** | 59.7 | 62.2 | 66.7 | 62.5 | 62.3 | 66.7 | 0.0 |
| 9.0 | 76.5 | 75.3 | 76.5 | 66.7 | 74.4 | 76.2 | **(*f_K_*)^3^** | 67.5 | 67.5 | 65.5 | 67.5 | 65.9 | 67.5 | 0.9 |
| 3.6 | 71.1 | 71.1 | 55.0 | 66.7 | 61.8 | 56.4 | **asin(*f_K_*)** | 53.8 | 54.8 | 66.7 | 55.4 | 46.2 | 66.7 | 0.0 |
| 0.0 | 74.2 | 51.6 | 50.7 | 66.7 | 67.4 | 74.2 | ***f_N_*** | 69.9 | 73.0 | 66.7 | 63.3 | 72.3 | 73.0 | 0.0 |
| -3.7 | 70.5 | 46.6 | 70.5 | 66.7 | 62.4 | 67.4 | **ln(*f_N_*)** | 60.5 | 20.4 | 66.7 | 67.4 | 74.1 | 74.1 | 1.1 |
| -6.7 | 67.5 | 54.3 | 49.2 | 66.7 | 61.4 | 67.5 | $\frac{\mathbf{1}}{\boldsymbol{f}_{\boldsymbol{N}}}$ | 65.0 | 39.6 | 64.4 | 54.3 | 72.5 | 72.5 | -0.5 |
| -4.4 | 69.8 | 56.7 | 63.3 | 66.7 | 65.3 | 69.8 | $\sqrt{\boldsymbol{f}_{\mathbf{N}}}$ | 64.9 | 43.3 | 66.7 | 75.0 | 74.7 | 75.0 | 2.0 |
| -0.8 | 73.3 | 50.0 | 47.4 | 66.7 | 68.1 | 73.3 | **(*f_N_*)^2^** | 67.5 | 74.4 | 66.7 | 68.4 | 72.3 | 74.4 | 1.4 |
| -8.3 | 65.9 | 62.2 | 52.1 | 65.5 | 65.9 | 63.4 | **(*f_N_*)^3^** | 67.5 | 69.6 | 66.7 | 69.1 | 75.6 | 75.6 | 2.6 |
| -1.9 | 72.3 | 55.4 | 70.6 | 65.5 | 70.3 | 72.3 | **asin(*f_N_*)** | 66.7 | 75.6 | 66.7 | 68.2 | 76.9 | 76.9 | 4.0 |
| 0.0 | 63.2 | 56.8 | 62.7 | 63.2 | 56.8 | 59.7 | ***f_R_*** | 63.3 | 16.3 | 66.7 | 54.3 | 54.1 | 66.7 | 0.0 |
| 3.4 | 66.7 | 54.1 | 47.6 | 66.7 | 58.1 | 57.1 | **ln(*f_R_*)** | 65.1 | 53.5 | 66.7 | 57.1 | 49.4 | 66.7 | 0.0 |
| 3.4 | 66.7 | 51.3 | 60.2 | 66.7 | 55.4 | 53.3 | $\frac{\mathbf{1}}{\boldsymbol{f}_{\boldsymbol{R}}}$ | 54.8 | 44.4 | 65.5 | 60.8 | 55.2 | 65.5 | -1.1 |
| 2.3 | 65.5 | 53.3 | 54.5 | 65.5 | 54.5 | 57.9 | $\sqrt{\boldsymbol{f}_{\mathbf{R}}}$ | 56.1 | 48.0 | 65.5 | 56.5 | 52.6 | 65.5 | -1.1 |
| 3.4 | 66.7 | 62.7 | 60.5 | 66.7 | 51.9 | 64.2 | **(*f_R_*)^2^** | 64.1 | 50.7 | 65.5 | 62.8 | 57.5 | 65.5 | -1.1 |
| 5.1 | 68.4 | 57.1 | 58.0 | 66.7 | 59.5 | 68.4 | **(*f_R_*)^3^** | 60.8 | 57.5 | 66.7 | 60.0 | 57.5 | 66.7 | 0.0 |
| 1.2 | 64.4 | 61.5 | 59.5 | 64.4 | 59.0 | 59.7 | **asin(*f_R_*)** | 51.9 | 52.1 | 66.7 | 58.8 | 53.3 | 66.7 | 0.0 |
| 0.0 | 73.0 | 56.0 | 71.1 | 66.7 | 60.2 | 73.0 | ***f_E_*** | 61.5 | 63.5 | 66.7 | 69.0 | 65.2 | 69.0 | 0.0 |
| -0.5 | 72.5 | 70.1 | 72.5 | 66.7 | 68.3 | 71.1 | **ln(*f_E_*)** | 63.4 | 55.8 | 66.7 | 49.3 | 65.9 | 66.7 | -2.4 |
| 4.8 | 77.8 | 65.8 | 77.8 | 66.7 | 67.4 | 72.7 | $\frac{\mathbf{1}}{\boldsymbol{f}_{\boldsymbol{E}}}$ | 55.3 | 50.0 | 66.7 | 65.9 | 61.5 | 66.7 | -2.4 |
| -3.0 | 70.0 | 62.5 | 70.0 | 66.7 | 67.5 | 69.3 | $\sqrt{\boldsymbol{f}_{\mathbf{E}}}$ | 54.5 | 54.4 | 65.5 | 64.2 | 65.2 | 65.5 | -3.5 |
| -0.2 | 72.7 | 60.8 | 71.3 | 65.5 | 61.2 | 72.7 | **(*f_E_*)^2^** | 60.5 | 58.1 | 66.7 | 58.7 | 71.1 | 71.1 | 2.1 |
| 0.2 | 73.2 | 56.8 | 58.0 | 66.7 | 53.7 | 73.2 | **(*f_E_*)^3^** | 57.1 | 76.9 | 66.7 | 67.4 | 73.3 | 76.9 | 7.9 |
| -0.5 | 72.5 | 66.7 | 72.5 | 66.7 | 65.9 | 71.1 | **asin(*f_E_*)** | 61.7 | 72.1 | 66.7 | 47.2 | 65.9 | 72.1 | 3.0 |

Table A.6 The classification of Normal beat versus PVC based on balanced data by RUS method. Unfiltered stands for the classification based on the raw ECG signal which don’t be processed by filter. Filtered stands for the classification based on the filtered ECG signal which is processed by a 0.5-30Hz 8^th^ order Butterworth bandpass filter. Max stands for the maximum F1 score of classifiers. D value stands for the difference value of maximum F1 score between original feature and transformed feature.

| **Unfiltered** | | | | | | | **Feature Transformation** | **Filtered** | | | | | | |
| --- | --- | --- | --- | --- | --- | --- | --- | --- | --- | --- | --- | --- | --- | --- |
| **D-value  (%)** | **Max  (%)** | **KNN  (%)** | **NN  (%)** | **SVM  (%)** | **TREE  (%)** | **Naïve Bayes (%)** |  | **KNN  (%)** | **NN  (%)** | **SVM  (%)** | **TREE  (%)** | **Naïve Bayes (%)** | **Max  (%)** | **D-value  (%)** |
| 0.0 | 66.7 | 60.5 | 61.4 | 66.7 | 40.6 | 60.0 | ***f_S_*** | 62.5 | 59.6 | 65.5 | 61.5 | 49.4 | 65.5 | 0.0 |
| 0.7 | 67.4 | 56.0 | 67.4 | 65.5 | 49.4 | 62.4 | **ln(*f_S_*)** | 62.5 | 54.5 | 64.3 | 58.7 | 52.1 | 64.3 | -1.2 |
| 7.8 | 74.4 | 69.3 | 57.4 | 66.7 | 73.4 | 74.4 | $\frac{\mathbf{1}}{\boldsymbol{f}_{\boldsymbol{S}}}$ | 61.2 | 53.8 | 64.3 | 62.2 | 50.7 | 64.3 | -1.2 |
| 0.0 | 66.7 | 52.9 | 24.6 | 66.7 | 40.6 | 58.7 | $\sqrt{\boldsymbol{f}_{\mathbf{S}}}$ | 60.0 | 54.1 | 66.7 | 65.8 | 55.2 | 66.7 | 1.2 |
| -2.3 | 64.3 | 60.2 | 58.2 | 64.3 | 51.4 | 59.3 | **(*f_S_*)^2^** | 67.5 | 60.3 | 65.5 | 59.5 | 43.2 | 67.5 | 2.0 |
| 0.0 | 66.7 | 64.9 | 59.3 | 66.7 | 61.5 | 63.9 | **(*f_S_*)^3^** | 69.4 | 63.0 | 66.7 | 71.4 | 45.3 | 71.4 | 5.9 |
| 0.9 | 67.6 | 56.0 | 56.3 | 66.7 | 52.1 | 67.6 | **asin(*f_S_*)** | 63.4 | 62.5 | 66.7 | 64.0 | 66.7 | 66.7 | 1.2 |
| 0.0 | 66.7 | 62.3 | 24.6 | 66.7 | 51.4 | 40.5 | ***f_Z_*** | 74.3 | 38.7 | 66.7 | 65.8 | 60.0 | 74.3 | 0.0 |
| 0.0 | 66.7 | 66.7 | NaN | 64.3 | 48.0 | 40.0 | **ln(*f_Z_*)** | 73.2 | 51.9 | 66.7 | 60.5 | 52.1 | 73.2 | -1.0 |
| 0.0 | 66.7 | 65.8 | 21.7 | 66.7 | 61.0 | 50.9 | $\frac{\mathbf{1}}{\boldsymbol{f}_{\boldsymbol{Z}}}$ | 72.2 | 53.2 | 66.7 | 65.9 | 44.8 | 72.2 | -2.1 |
| 0.0 | 66.7 | 64.0 | 34.4 | 66.7 | 52.6 | 35.9 | $\sqrt{\boldsymbol{f}_{\mathbf{Z}}}$ | 73.2 | 54.5 | 66.7 | 68.1 | 48.6 | 73.2 | -1.0 |
| 0.0 | 66.7 | 63.2 | 47.5 | 66.7 | 60.0 | 48.2 | **(*f_Z_*)^2^** | 74.0 | 62.2 | 64.3 | 63.3 | 60.7 | 74.0 | -0.3 |
| 0.0 | 66.7 | 65.8 | 40.0 | 66.7 | 64.2 | 46.9 | **(*f_Z_*)^3^** | 76.7 | 63.6 | 66.7 | 75.6 | 63.8 | 76.7 | 2.4 |
| 0.0 | 66.7 | 62.3 | 29.4 | 66.7 | 51.4 | 40.5 | **asin(*f_Z_*)** | 74.3 | 57.1 | 66.7 | 65.8 | 60.0 | 74.3 | 0.0 |
| 0.0 | 65.5 | 57.5 | 40.6 | 65.5 | 51.2 | 33.3 | ***f_K_*** | 51.4 | 56.2 | 65.5 | 54.5 | 47.5 | 65.5 | 0.0 |
| 0.0 | 65.5 | 53.2 | 40.5 | 65.5 | 47.6 | 42.9 | **ln(*f_K_*)** | 58.7 | 45.7 | 65.5 | 61.9 | 28.1 | 65.5 | 0.0 |
| 0.0 | 65.5 | 61.0 | 57.1 | 65.5 | 54.5 | 48.0 | $\frac{\mathbf{1}}{\boldsymbol{f}_{\boldsymbol{K}}}$ | 55.3 | 38.8 | 66.7 | 58.8 | 41.8 | 66.7 | 1.2 |
| 1.2 | 66.7 | 50.0 | 40.0 | 66.7 | 54.8 | 33.3 | $\sqrt{\boldsymbol{f}_{\mathbf{K}}}$ | 51.4 | 44.8 | 66.7 | 58.5 | 32.8 | 66.7 | 1.2 |
| 0.0 | 65.5 | 59.8 | 50.0 | 65.5 | 59.5 | 31.0 | **(*f_K_*)^2^** | 48.0 | 49.3 | 66.7 | 55.7 | 50.5 | 66.7 | 1.2 |
| 4.6 | 70.1 | 68.2 | 52.3 | 66.7 | 70.1 | 23.1 | **(*f_K_*)^3^** | 49.3 | 62.1 | 66.7 | 57.9 | 59.3 | 66.7 | 1.2 |
| 0.0 | 65.5 | 52.6 | 50.0 | 65.5 | 52.9 | 41.2 | **asin(*f_K_*)** | 58.7 | 48.6 | 65.5 | 61.9 | 28.1 | 65.5 | 0.0 |
| 0.0 | 66.7 | 54.3 | 46.9 | 66.7 | 45.0 | 60.0 | ***f_N_*** | 45.9 | 50.0 | 66.7 | 51.2 | 64.3 | 66.7 | 0.0 |
| 0.0 | 66.7 | 51.8 | 51.4 | 66.7 | 35.5 | 58.7 | **ln(*f_N_*)** | 48.1 | 69.6 | 66.7 | 44.7 | 64.0 | 69.6 | 2.9 |
| 0.0 | 66.7 | 53.7 | 43.0 | 66.7 | 48.6 | 50.7 | $\frac{\mathbf{1}}{\boldsymbol{f}_{\boldsymbol{N}}}$ | 57.1 | 31.9 | 66.7 | 66.7 | 63.9 | 66.7 | 0.0 |
| 0.0 | 66.7 | 49.4 | 29.2 | 66.7 | 46.8 | 57.9 | $\sqrt{\boldsymbol{f}_{\mathbf{N}}}$ | 48.0 | 54.5 | 66.7 | 51.9 | 60.2 | 66.7 | 0.0 |
| 0.0 | 66.7 | 42.7 | 61.4 | 66.7 | 50.0 | 58.8 | **(*f_N_*)^2^** | 50.6 | 58.5 | 66.7 | 38.9 | 60.5 | 66.7 | 0.0 |
| 0.0 | 66.7 | 52.5 | 57.5 | 66.7 | 53.5 | 59.1 | **(*f_N_*)^3^** | 48.0 | 58.4 | 66.7 | 54.3 | 57.8 | 66.7 | 0.0 |
| 0.0 | 66.7 | 48.8 | 57.5 | 66.7 | 47.2 | 59.5 | **asin(*f_N_*)** | 50.0 | 68.1 | 66.7 | 51.3 | 59.3 | 68.1 | 1.5 |
| 0.0 | 66.7 | 59.3 | 53.8 | 66.7 | 57.5 | 39.4 | ***f_R_*** | 60.7 | 50.6 | 66.7 | 46.4 | 47.8 | 66.7 | 0.0 |
| 0.0 | 66.7 | 56.8 | 35.7 | 66.7 | 62.5 | 37.9 | **ln(*f_R_*)** | 53.5 | 39.4 | 65.5 | 39.4 | 48.6 | 65.5 | -1.2 |
| 0.0 | 66.7 | 63.4 | 64.2 | 66.7 | 64.0 | 46.9 | $\frac{\mathbf{1}}{\boldsymbol{f}_{\boldsymbol{R}}}$ | 50.0 | 48.0 | 64.3 | 61.5 | 48.0 | 64.3 | -2.3 |
| -1.2 | 65.5 | 63.3 | 59.3 | 65.5 | 56.3 | 40.7 | $\sqrt{\boldsymbol{f}_{\mathbf{R}}}$ | 54.8 | 62.9 | 66.7 | 51.4 | 47.8 | 66.7 | 0.0 |
| 1.6 | 68.2 | 56.3 | 55.4 | 66.7 | 68.2 | 56.0 | **(*f_R_*)^2^** | 58.2 | 46.4 | 66.7 | 50.0 | 46.4 | 66.7 | 0.0 |
| 0.0 | 66.7 | 58.3 | 42.7 | 66.7 | 58.7 | 62.8 | **(*f_R_*)^3^** | 62.5 | 51.4 | 64.3 | 61.0 | 47.1 | 64.3 | -2.3 |
| -1.2 | 65.5 | 56.0 | 45.7 | 65.5 | 61.7 | 39.4 | **asin(*f_R_*)** | 60.0 | 44.1 | 66.7 | 51.4 | 47.1 | 66.7 | 0.0 |
| 0.0 | 66.7 | 66.7 | 55.0 | 66.7 | 64.3 | 61.2 | ***f_E_*** | 57.1 | 52.5 | 65.5 | 61.7 | 56.8 | 65.5 | 0.0 |
| 0.7 | 67.4 | 54.8 | 67.4 | 66.7 | 65.2 | 62.8 | **ln(*f_E_*)** | 56.8 | 67.4 | 66.7 | 57.1 | 65.2 | 67.4 | 1.9 |
| 0.0 | 66.7 | 51.3 | 66.7 | 66.7 | 58.5 | 65.2 | $\frac{\mathbf{1}}{\boldsymbol{f}_{\boldsymbol{E}}}$ | 56.3 | 63.7 | 65.5 | 64.4 | 66.0 | 66.0 | 0.4 |
| 3.2 | 69.9 | 57.1 | 69.9 | 66.7 | 56.1 | 61.2 | $\sqrt{\boldsymbol{f}_{\mathbf{E}}}$ | 58.2 | 47.5 | 66.7 | 57.5 | 63.5 | 66.7 | 1.2 |
| 0.8 | 67.5 | 55.6 | 67.5 | 66.7 | 59.0 | 54.5 | **(*f_E_*)^2^** | 53.9 | 65.9 | 65.5 | 52.1 | 40.6 | 65.9 | 0.4 |
| 0.0 | 66.7 | 58.7 | 62.7 | 66.7 | 54.8 | 52.3 | **(*f_E_*)^3^** | 50.0 | 69.2 | 66.7 | 55.7 | 35.8 | 69.2 | 3.7 |
| 3.8 | 70.5 | 54.8 | 70.5 | 66.7 | 66.7 | 62.8 | **asin(*f_E_*)** | 56.8 | 61.2 | 65.5 | 57.1 | 65.2 | 65.5 | 0.0 |

Table A.7 The classification of Normal beat versus APB based on balanced data by SMOTE method. Unfiltered stands for the classification based on the raw ECG signal which don’t be processed by filter. Filtered stands for the classification based on the filtered ECG signal which is processed by a 0.5-30Hz 8^th^ order Butterworth bandpass filter. Max stands for the maximum F1 score of classifiers. D value stands for the difference value of maximum F1 score between original feature and transformed feature.

| **Unfiltered** | | | | | | | **Feature Transformation** | **Filtered** | | | | | | |
| --- | --- | --- | --- | --- | --- | --- | --- | --- | --- | --- | --- | --- | --- | --- |
| **D-value  (%)** | **Max  (%)** | **KNN  (%)** | **NN  (%)** | **SVM  (%)** | **TREE  (%)** | **Naïve Bayes (%)** |  | **KNN  (%)** | **NN  (%)** | **SVM  (%)** | **TREE  (%)** | **Naïve Bayes (%)** | **Max  (%)** | **D-value  (%)** |
| 0.0 | 90.3 | 90.3 | 71.2 | 45.8 | 86.4 | 69.2 | ***f_S_*** | 87.7 | 57.3 | 47.2 | 78.0 | 63.5 | 87.7 | 0.0 |
| -0.5 | 89.8 | 89.8 | 64.6 | 24.8 | 87.2 | 67.7 | **ln(*f_S_*)** | 88.2 | 70.2 | 23.6 | 84.1 | 74.2 | 88.2 | 0.5 |
| 3.8 | 94.0 | 94.0 | 84.4 | 4.5 | 91.1 | 89.7 | $\frac{\mathbf{1}}{\boldsymbol{f}_{\boldsymbol{S}}}$ | 91.3 | 68.0 | 12.6 | 86.3 | 77.8 | 91.3 | 3.6 |
| -2.5 | 87.8 | 87.8 | 68.2 | 45.6 | 81.3 | 70.4 | $\sqrt{\boldsymbol{f}_{\mathbf{S}}}$ | 86.7 | 64.2 | 49.7 | 81.0 | 66.3 | 86.7 | -1.0 |
| 1.0 | 91.3 | 91.3 | 74.7 | 58.1 | 81.2 | 72.0 | **(*f_S_*)^2^** | 85.2 | 65.9 | 55.3 | 76.3 | 61.0 | 85.2 | -2.5 |
| 3.2 | 93.5 | 93.5 | 74.3 | 46.9 | 86.6 | 76.9 | **(*f_S_*)^3^** | 86.2 | 64.8 | 38.1 | 81.8 | 64.2 | 86.2 | -1.5 |
| -4.4 | 85.9 | 85.9 | 69.6 | 50.6 | 83.8 | 73.2 | **asin(*f_S_*)** | 87.7 | 66.7 | 56.2 | 85.7 | 70.5 | 87.7 | 0.0 |
| 0.0 | 92.4 | 92.4 | 38.1 | 61.5 | 76.5 | 60.4 | ***f_Z_*** | 94.6 | 66.3 | 29.3 | 87.7 | 65.9 | 94.6 | 0.0 |
| 3.9 | 96.3 | 96.3 | 48.1 | 18.9 | 86.7 | 51.1 | **ln(*f_Z_*)** | 94.6 | 67.1 | 38.5 | 85.2 | 67.1 | 94.6 | 0.0 |
| 5.7 | 98.1 | 98.1 | 62.6 | 4.6 | 87.6 | 69.6 | $\frac{\mathbf{1}}{\boldsymbol{f}_{\boldsymbol{Z}}}$ | 94.6 | 67.4 | 60.8 | 84.0 | 66.7 | 94.6 | 0.0 |
| 0.0 | 92.4 | 92.4 | 58.8 | 16.3 | 75.8 | 48.7 | $\sqrt{\boldsymbol{f}_{\mathbf{Z}}}$ | 95.2 | 66.2 | 68.0 | 87.1 | 66.3 | 95.2 | 0.6 |
| 1.7 | 94.0 | 94.0 | 31.1 | 62.4 | 86.4 | 69.1 | **(*f_Z_*)^2^** | 94.0 | 67.9 | 19.1 | 86.3 | 68.2 | 94.0 | -0.6 |
| 1.1 | 93.5 | 93.5 | 64.1 | 16.5 | 79.8 | 70.1 | **(*f_Z_*)^3^** | 95.2 | 70.8 | 66.4 | 92.1 | 70.1 | 95.2 | 0.6 |
| 0.0 | 92.4 | 92.4 | 53.4 | 60.6 | 76.5 | 60.4 | **asin(*f_Z_*)** | 94.6 | 68.5 | 64.3 | 87.7 | 65.9 | 94.6 | 0.0 |
| 0.0 | 86.3 | 86.3 | 60.6 | 41.3 | 73.7 | 64.7 | ***f_K_*** | 88.8 | 64.0 | 50.3 | 77.1 | 58.1 | 88.8 | 0.0 |
| 1.4 | 87.8 | 87.8 | 72.0 | 41.1 | 78.0 | 64.6 | **ln(*f_K_*)** | 88.8 | 68.6 | 42.5 | 86.7 | 63.2 | 88.8 | 0.0 |
| 0.5 | 86.8 | 86.8 | 63.6 | 45.7 | 77.3 | 65.9 | $\frac{\mathbf{1}}{\boldsymbol{f}_{\boldsymbol{K}}}$ | 91.3 | 75.3 | 43.4 | 82.6 | 69.0 | 91.3 | 2.6 |
| 1.9 | 88.3 | 88.3 | 66.7 | 55.1 | 76.7 | 65.5 | $\sqrt{\boldsymbol{f}_{\mathbf{K}}}$ | 92.4 | 53.7 | 49.1 | 84.0 | 62.3 | 92.4 | 3.6 |
| 1.0 | 87.3 | 87.3 | 61.6 | 57.0 | 73.8 | 59.9 | **(*f_K_*)^2^** | 90.3 | 64.2 | 20.5 | 81.0 | 60.6 | 90.3 | 1.5 |
| 0.5 | 86.8 | 86.8 | 63.4 | 62.1 | 81.9 | 62.8 | **(*f_K_*)^3^** | 88.8 | 60.3 | 67.9 | 72.7 | 63.2 | 88.8 | 0.0 |
| 1.0 | 87.3 | 87.3 | 63.4 | 40.0 | 80.0 | 65.5 | **asin(*f_K_*)** | 88.8 | 69.9 | 44.0 | 81.2 | 63.2 | 88.8 | 0.0 |
| 0.0 | 82.7 | 82.7 | 66.1 | 50.0 | 76.3 | 62.5 | ***f_N_*** | 88.3 | 48.5 | 12.5 | 76.3 | 59.8 | 88.3 | 0.0 |
| 5.1 | 87.8 | 87.8 | 58.5 | 59.9 | 79.0 | 51.3 | **ln(*f_N_*)** | 87.3 | 62.9 | 39.0 | 74.6 | 57.1 | 87.3 | -1.0 |
| 8.1 | 90.8 | 90.8 | 74.3 | NaN | 86.4 | 74.3 | $\frac{\mathbf{1}}{\boldsymbol{f}_{\boldsymbol{N}}}$ | 90.8 | 72.1 | 61.0 | 79.8 | 73.0 | 90.8 | 2.5 |
| 3.6 | 86.3 | 86.3 | 64.2 | 23.5 | 80.3 | 68.1 | $\sqrt{\boldsymbol{f}_{\mathbf{N}}}$ | 88.8 | 53.2 | 61.9 | 80.5 | 60.1 | 88.8 | 0.5 |
| 5.5 | 88.3 | 88.3 | 64.1 | 65.1 | 83.2 | 64.6 | **(*f_N_*)^2^** | 85.9 | 58.5 | 66.1 | 74.4 | 59.4 | 85.9 | -2.4 |
| 6.5 | 89.3 | 89.3 | 64.7 | 67.4 | 85.9 | 76.8 | **(*f_N_*)^3^** | 86.3 | 66.7 | 66.7 | 82.2 | 74.2 | 86.3 | -1.9 |
| 4.1 | 86.8 | 86.8 | 31.3 | 25.0 | 82.5 | 67.8 | **asin(*f_N_*)** | 90.8 | 64.5 | 55.1 | 83.1 | 64.3 | 90.8 | 2.5 |
| 0.0 | 87.3 | 87.3 | 72.1 | 58.4 | 75.7 | 71.5 | ***f_R_*** | 86.3 | 69.2 | 65.4 | 74.5 | 70.1 | 86.3 | 0.0 |
| 2.0 | 89.3 | 89.3 | 70.7 | 37.1 | 78.0 | 69.3 | **ln(*f_R_*)** | 87.8 | 72.2 | 69.0 | 80.5 | 74.9 | 87.8 | 1.4 |
| 4.0 | 91.3 | 91.3 | 74.7 | 62.7 | 86.6 | 73.6 | $\frac{\mathbf{1}}{\boldsymbol{f}_{\boldsymbol{R}}}$ | 89.8 | 71.7 | 66.0 | 82.1 | 75.5 | 89.8 | 3.4 |
| 0.0 | 87.3 | 87.3 | 73.1 | 62.2 | 79.0 | 70.8 | $\sqrt{\boldsymbol{f}_{\mathbf{R}}}$ | 89.8 | 64.2 | 64.2 | 84.1 | 73.5 | 89.8 | 3.4 |
| 3.0 | 90.3 | 90.3 | 72.8 | 58.9 | 78.5 | 67.8 | **(*f_R_*)^2^** | 84.0 | 63.4 | 63.8 | 72.4 | 69.5 | 84.0 | -2.3 |
| -1.4 | 85.9 | 85.9 | 70.7 | 58.5 | 72.8 | 65.5 | **(*f_R_*)^3^** | 85.4 | 70.3 | 65.4 | 71.1 | 68.1 | 85.4 | -0.9 |
| 0.5 | 87.8 | 87.8 | 71.3 | 57.8 | 77.6 | 71.5 | **asin(*f_R_*)** | 86.8 | 67.1 | 65.4 | 75.5 | 70.7 | 86.8 | 0.5 |
| 0.0 | 87.3 | 87.3 | 71.0 | 53.9 | 73.4 | 67.9 | ***f_E_*** | 86.8 | 65.5 | 38.9 | 77.2 | 57.3 | 86.8 | 0.0 |
| 2.0 | 89.3 | 89.3 | 72.9 | 48.3 | 79.5 | 76.3 | **ln(*f_E_*)** | 87.8 | 65.3 | 59.9 | 82.6 | 61.2 | 87.8 | 1.0 |
| -0.1 | 87.2 | 87.2 | 69.7 | 42.7 | 80.9 | 77.1 | $\frac{\mathbf{1}}{\boldsymbol{f}_{\boldsymbol{E}}}$ | 86.8 | 71.8 | 36.1 | 83.0 | 72.6 | 86.8 | 0.0 |
| 3.0 | 90.3 | 90.3 | 57.5 | 54.9 | 79.0 | 70.7 | $\sqrt{\boldsymbol{f}_{\mathbf{E}}}$ | 84.9 | 58.8 | 38.9 | 74.1 | 60.3 | 84.9 | -1.9 |
| 3.0 | 90.3 | 90.3 | 75.7 | 45.3 | 79.3 | 63.1 | **(*f_E_*)^2^** | 86.8 | 60.9 | 59.4 | 82.1 | 54.0 | 86.8 | 0.0 |
| -0.6 | 86.7 | 86.7 | 73.4 | 46.9 | 77.8 | 55.2 | **(*f_E_*)^3^** | 83.6 | 59.7 | 55.9 | 77.6 | 57.1 | 83.6 | -3.2 |
| 2.0 | 89.3 | 89.3 | 64.3 | 55.1 | 79.3 | 76.3 | **asin(*f_E_*)** | 86.8 | 69.3 | 63.7 | 83.3 | 61.2 | 86.8 | 0.0 |

Table A.8 The classification of Normal beat versus AF based on balanced data by SMOTE method. Unfiltered stands for the classification based on the raw ECG signal which don’t be processed by filter. Filtered stands for the classification based on the filtered ECG signal which is processed by a 0.5-30Hz 8^th^ order Butterworth bandpass filter. Max stands for the maximum F1 score of classifiers. D value stands for the difference value of maximum F1 score between original feature and transformed feature.

| **Unfiltered** | | | | | | | **Feature Transformation** | **Filtered** | | | | | | |
| --- | --- | --- | --- | --- | --- | --- | --- | --- | --- | --- | --- | --- | --- | --- |
| **D-value  (%)** | **Max  (%)** | **KNN  (%)** | **NN  (%)** | **SVM  (%)** | **TREE  (%)** | **Naïve Bayes (%)** |  | **KNN  (%)** | **NN  (%)** | **SVM  (%)** | **TREE  (%)** | **Naïve Bayes (%)** | **Max  (%)** | **D-value  (%)** |
| 0.0 | 75.6 | 75.6 | 61.6 | 67.5 | 67.5 | 60.9 | ***f_S_*** | 71.6 | 65.5 | 66.9 | 64.0 | 64.4 | 71.6 | 0.0 |
| 0.2 | 75.8 | 75.8 | 54.3 | 67.5 | 61.7 | 63.8 | **ln(*f_S_*)** | 70.5 | 67.4 | 67.5 | 67.1 | 66.3 | 70.5 | -1.1 |
| 4.6 | 80.2 | 80.2 | 73.1 | 66.9 | 75.9 | 79.5 | $\frac{\mathbf{1}}{\boldsymbol{f}_{\boldsymbol{S}}}$ | 91.9 | 76.5 | 66.4 | 90.0 | 89.0 | 91.9 | 20.3 |
| -3.4 | 72.2 | 72.2 | 63.2 | 66.9 | 62.4 | 62.6 | $\sqrt{\boldsymbol{f}_{\mathbf{S}}}$ | 75.6 | 60.9 | 66.9 | 67.9 | 65.5 | 75.6 | 4.0 |
| -2.3 | 73.3 | 73.3 | 62.9 | 66.9 | 65.9 | 64.5 | **(*f_S_*)^2^** | 73.3 | 57.9 | 67.5 | 68.9 | 64.4 | 73.3 | 1.7 |
| 3.3 | 78.9 | 78.9 | 49.4 | 66.9 | 70.1 | 67.5 | **(*f_S_*)^3^** | 76.0 | 64.5 | 67.5 | 67.1 | 65.9 | 76.0 | 4.4 |
| -2.6 | 73.0 | 73.0 | 66.3 | 66.9 | 62.0 | 63.9 | **asin(*f_S_*)** | 69.0 | 62.3 | 67.5 | 61.7 | 66.7 | 69.0 | -2.6 |
| 0.0 | 83.3 | 83.3 | 45.3 | 67.5 | 76.7 | 57.9 | ***f_Z_*** | 96.3 | 56.7 | 66.9 | 83.0 | 66.4 | 96.3 | 0.0 |
| 0.9 | 84.3 | 84.3 | 59.5 | 67.5 | 78.6 | 57.0 | **ln(*f_Z_*)** | 95.8 | 52.3 | 66.4 | 83.0 | 66.7 | 95.8 | -0.6 |
| 7.0 | 90.4 | 90.4 | 56.4 | 66.9 | 76.8 | 61.1 | $\frac{\mathbf{1}}{\boldsymbol{f}_{\boldsymbol{Z}}}$ | 96.3 | 45.2 | 66.4 | 82.0 | 66.7 | 96.3 | 0.0 |
| 7.0 | 90.4 | 90.4 | 56.1 | 67.5 | 77.5 | 54.8 | $\sqrt{\boldsymbol{f}_{\mathbf{Z}}}$ | 95.8 | 66.7 | 65.9 | 84.0 | 65.5 | 95.8 | -0.6 |
| -1.1 | 82.3 | 82.3 | 54.4 | 67.5 | 72.1 | 59.4 | **(*f_Z_*)^2^** | 97.0 | 52.2 | 66.9 | 82.2 | 66.7 | 97.0 | 0.7 |
| 0.4 | 83.8 | 83.8 | 61.0 | 67.5 | 75.4 | 63.3 | **(*f_Z_*)^3^** | 96.4 | 63.6 | 66.4 | 85.9 | 68.2 | 96.4 | 0.1 |
| 0.0 | 83.3 | 83.3 | 55.4 | 66.9 | 76.7 | 57.9 | **asin(*f_Z_*)** | 96.3 | 57.1 | 67.5 | 83.0 | 66.4 | 96.3 | 0.0 |
| 0.0 | 81.1 | 81.1 | 74.7 | 67.5 | 76.3 | 73.3 | ***f_K_*** | 78.5 | 68.8 | 66.9 | 68.0 | 73.7 | 78.5 | 0.0 |
| -1.8 | 79.3 | 79.3 | 63.6 | 66.4 | 72.3 | 70.4 | **ln(*f_K_*)** | 78.0 | 59.8 | 67.5 | 62.3 | 69.2 | 78.0 | -0.5 |
| -1.8 | 79.3 | 79.3 | 71.7 | 67.5 | 69.9 | 67.9 | $\frac{\mathbf{1}}{\boldsymbol{f}_{\boldsymbol{K}}}$ | 81.1 | 59.8 | 67.5 | 65.8 | 64.6 | 81.1 | 2.6 |
| -0.4 | 80.7 | 80.7 | 71.9 | 67.5 | 71.5 | 73.0 | $\sqrt{\boldsymbol{f}_{\mathbf{K}}}$ | 82.8 | 65.4 | 67.5 | 68.9 | 69.9 | 82.8 | 4.3 |
| 3.6 | 84.7 | 84.7 | 75.4 | 66.9 | 80.2 | 76.7 | **(*f_K_*)^2^** | 82.1 | 74.8 | 67.5 | 69.5 | 72.1 | 82.1 | 3.7 |
| 1.8 | 83.0 | 83.0 | 76.8 | 67.5 | 78.8 | 77.3 | **(*f_K_*)^3^** | 81.7 | 78.2 | 66.4 | 75.0 | 73.2 | 81.7 | 3.2 |
| 0.0 | 81.1 | 81.1 | 63.8 | 67.5 | 72.9 | 70.4 | **asin(*f_K_*)** | 80.7 | 62.7 | 67.5 | 68.3 | 68.5 | 80.7 | 2.2 |
| 0.0 | 76.9 | 76.9 | 69.6 | 67.5 | 70.5 | 73.4 | ***f_N_*** | 82.1 | 72.4 | 67.5 | 80.0 | 82.0 | 82.1 | 0.0 |
| -0.3 | 76.7 | 76.7 | 60.1 | 67.5 | 64.9 | 58.1 | **ln(*f_N_*)** | 76.6 | 61.0 | 67.5 | 69.0 | 65.7 | 76.6 | -5.6 |
| 1.9 | 78.8 | 78.8 | 66.3 | 66.4 | 71.6 | 69.1 | $\frac{\mathbf{1}}{\boldsymbol{f}_{\boldsymbol{N}}}$ | 84.6 | 58.2 | 67.5 | 74.4 | 75.5 | 84.6 | 2.5 |
| 3.3 | 80.2 | 80.2 | 65.0 | 66.9 | 73.9 | 73.1 | $\sqrt{\boldsymbol{f}_{\mathbf{N}}}$ | 77.5 | 80.7 | 65.9 | 73.3 | 79.7 | 80.7 | -1.4 |
| 1.0 | 77.9 | 77.9 | 69.1 | 67.5 | 74.1 | 72.6 | **(*f_N_*)^2^** | 79.3 | 62.8 | 67.5 | 73.1 | 81.9 | 81.9 | -0.2 |
| 6.5 | 83.4 | 83.4 | 69.8 | 66.4 | 79.5 | 76.0 | **(*f_N_*)^3^** | 84.3 | 70.3 | 67.5 | 80.5 | 78.0 | 84.3 | 2.1 |
| 5.2 | 82.1 | 82.1 | 69.9 | 66.9 | 75.6 | 73.3 | **asin(*f_N_*)** | 81.6 | 82.2 | 67.5 | 72.6 | 79.7 | 82.2 | 0.1 |
| 0.0 | 67.9 | 67.9 | 59.6 | 66.4 | 63.2 | 57.7 | ***f_R_*** | 79.3 | 47.4 | 67.5 | 64.5 | 51.9 | 79.3 | 0.0 |
| 1.5 | 69.4 | 69.4 | 51.9 | 67.5 | 62.1 | 55.9 | **ln(*f_R_*)** | 74.7 | 54.0 | 67.5 | 64.7 | 50.6 | 74.7 | -4.6 |
| 4.3 | 72.2 | 72.2 | 54.4 | 67.5 | 63.5 | 53.6 | $\frac{\mathbf{1}}{\boldsymbol{f}_{\boldsymbol{R}}}$ | 75.1 | 50.9 | 67.5 | 59.0 | 54.0 | 75.1 | -4.2 |
| 3.6 | 71.5 | 71.5 | 56.1 | 67.5 | 63.1 | 58.0 | $\sqrt{\boldsymbol{f}_{\mathbf{R}}}$ | 76.3 | 51.9 | 67.5 | 58.7 | 49.7 | 76.3 | -3.0 |
| 3.3 | 71.2 | 71.2 | 60.6 | 67.5 | 64.3 | 62.1 | **(*f_R_*)^2^** | 77.7 | 54.1 | 67.5 | 65.9 | 60.6 | 77.7 | -1.7 |
| 5.8 | 73.8 | 73.8 | 63.2 | 66.9 | 69.4 | 64.0 | **(*f_R_*)^3^** | 77.2 | 55.8 | 67.5 | 62.6 | 62.2 | 77.2 | -2.1 |
| -0.4 | 67.5 | 66.3 | 55.9 | 67.5 | 60.1 | 56.1 | **asin(*f_R_*)** | 77.7 | 55.2 | 67.5 | 62.7 | 55.5 | 77.7 | -1.6 |
| 0.0 | 79.8 | 79.8 | 66.7 | 67.5 | 69.5 | 65.4 | ***f_E_*** | 71.2 | 61.0 | 67.5 | 59.2 | 50.9 | 71.2 | 0.0 |
| -0.9 | 78.9 | 78.9 | 67.6 | 65.3 | 69.3 | 68.0 | **ln(*f_E_*)** | 77.5 | 48.5 | 67.5 | 66.3 | 46.4 | 77.5 | 6.3 |
| 2.1 | 81.9 | 81.9 | 73.2 | 67.5 | 69.9 | 72.2 | $\frac{\mathbf{1}}{\boldsymbol{f}_{\boldsymbol{E}}}$ | 77.8 | 57.3 | 66.4 | 68.9 | 45.4 | 77.8 | 6.6 |
| 4.3 | 84.0 | 84.0 | 68.9 | 66.9 | 70.7 | 68.9 | $\sqrt{\boldsymbol{f}_{\mathbf{E}}}$ | 76.2 | 54.7 | 66.4 | 65.8 | 48.2 | 76.2 | 5.1 |
| 3.0 | 82.8 | 82.8 | 65.9 | 66.9 | 72.2 | 66.7 | **(*f_E_*)^2^** | 76.1 | 51.7 | 67.5 | 64.3 | 57.6 | 76.1 | 5.0 |
| 0.0 | 79.8 | 79.8 | 61.4 | 67.5 | 71.5 | 65.1 | **(*f_E_*)^3^** | 75.1 | 65.5 | 67.5 | 70.2 | 62.0 | 75.1 | 4.0 |
| -0.9 | 78.9 | 78.9 | 68.5 | 65.3 | 69.0 | 68.0 | **asin(*f_E_*)** | 77.1 | 51.2 | 67.5 | 66.7 | 46.4 | 77.1 | 5.9 |

Table A.9 The classification of Normal beat versus PVC based on balanced data by SMOTE method. Unfiltered stands for the classification based on the raw ECG signal which don’t be processed by filter. Filtered stands for the classification based on the filtered ECG signal which is processed by a 0.5-30Hz 8^th^ order Butterworth bandpass filter. Max stands for the maximum F1 score of classifiers. D value stands for the difference value of maximum F1 score between original feature and transformed feature.

| **Unfiltered** | | | | | | | **Feature Transformation** | **Filtered** | | | | | | |
| --- | --- | --- | --- | --- | --- | --- | --- | --- | --- | --- | --- | --- | --- | --- |
| **D-value  (%)** | **Max  (%)** | **KNN  (%)** | **NN  (%)** | **SVM  (%)** | **TREE  (%)** | **Naïve Bayes (%)** |  | **KNN  (%)** | **NN  (%)** | **SVM  (%)** | **TREE  (%)** | **Naïve Bayes (%)** | **Max  (%)** | **D-value  (%)** |
| 0.0 | 77.5 | 77.5 | 54.9 | 67.5 | 67.4 | 59.6 | ***f_S_*** | 74.7 | 65.9 | 67.5 | 61.6 | 64.7 | 74.7 | 0.0 |
| -1.0 | 76.6 | 76.6 | 61.8 | 67.5 | 75.2 | 67.3 | **ln(*f_S_*)** | 74.3 | 64.9 | 67.5 | 63.8 | 66.3 | 74.3 | -0.4 |
| 5.4 | 83.0 | 83.0 | 57.1 | 68.0 | 75.6 | 76.3 | $\frac{\mathbf{1}}{\boldsymbol{f}_{\boldsymbol{S}}}$ | 78.5 | 56.7 | 66.9 | 73.4 | 52.4 | 78.5 | 3.8 |
| -2.9 | 74.6 | 74.6 | 53.0 | 68.0 | 63.5 | 55.1 | $\sqrt{\boldsymbol{f}_{\mathbf{S}}}$ | 77.2 | 57.9 | 68.0 | 71.3 | 65.8 | 77.2 | 2.5 |
| 2.7 | 80.2 | 80.2 | 55.6 | 68.0 | 65.9 | 62.7 | **(*f_S_*)^2^** | 78.4 | 54.5 | 68.0 | 70.7 | 68.8 | 78.4 | 3.7 |
| 4.4 | 82.0 | 82.0 | 53.8 | 66.9 | 66.7 | 64.7 | **(*f_S_*)^3^** | 84.0 | 71.4 | 68.0 | 79.0 | 51.9 | 84.0 | 9.3 |
| -1.3 | 76.2 | 76.2 | 47.1 | 66.9 | 66.3 | 56.3 | **asin(*f_S_*)** | 81.6 | 59.0 | 66.9 | 71.6 | 64.9 | 81.6 | 6.9 |
| 0.0 | 80.3 | 80.3 | 30.0 | 68.0 | 68.5 | 53.4 | ***f_Z_*** | 92.2 | 61.5 | 68.0 | 79.8 | 62.8 | 92.2 | 0.0 |
| 1.5 | 81.8 | 81.8 | 49.7 | 68.0 | 66.7 | 55.3 | **ln(*f_Z_*)** | 92.0 | 57.5 | 68.0 | 80.5 | 55.3 | 92.0 | -0.2 |
| 4.2 | 84.4 | 84.4 | 57.6 | 67.5 | 61.0 | 42.7 | $\frac{\mathbf{1}}{\boldsymbol{f}_{\boldsymbol{Z}}}$ | 92.6 | 56.8 | 68.0 | 83.6 | 50.0 | 92.6 | 0.4 |
| -0.5 | 79.7 | 79.7 | 59.1 | 68.0 | 70.1 | 55.3 | $\sqrt{\boldsymbol{f}_{\mathbf{Z}}}$ | 92.2 | 58.9 | 68.0 | 77.8 | 57.5 | 92.2 | 0.0 |
| 3.1 | 83.3 | 83.3 | 39.7 | 68.0 | 66.7 | 43.0 | **(*f_Z_*)^2^** | 94.5 | 70.0 | 68.0 | 82.1 | 63.3 | 94.5 | 2.3 |
| 3.6 | 83.9 | 83.9 | 54.0 | 68.0 | 65.5 | 42.9 | **(*f_Z_*)^3^** | 94.5 | 63.5 | 67.5 | 87.8 | 70.4 | 94.5 | 2.3 |
| 0.0 | 80.3 | 80.3 | 27.1 | 67.5 | 68.5 | 53.4 | **asin(*f_Z_*)** | 92.2 | 64.2 | 68.0 | 79.8 | 62.8 | 92.2 | 0.0 |
| 0.0 | 71.3 | 71.3 | 46.5 | 68.0 | 64.7 | 63.9 | ***f_K_*** | 73.9 | 43.2 | 66.9 | 62.4 | 59.6 | 73.9 | 0.0 |
| -2.6 | 68.7 | 68.7 | 43.2 | 68.0 | 65.8 | 55.6 | **ln(*f_K_*)** | 72.8 | 58.0 | 67.5 | 64.5 | 68.3 | 72.8 | -1.0 |
| -3.2 | 68.0 | 65.9 | 60.8 | 68.0 | 60.8 | 60.5 | $\frac{\mathbf{1}}{\boldsymbol{f}_{\boldsymbol{K}}}$ | 72.6 | 69.5 | 68.0 | 64.9 | 68.5 | 72.6 | -1.2 |
| 0.5 | 71.8 | 71.8 | 55.5 | 67.5 | 55.3 | 63.9 | $\sqrt{\boldsymbol{f}_{\mathbf{K}}}$ | 73.9 | 56.3 | 67.5 | 61.1 | 66.7 | 73.9 | 0.0 |
| 3.8 | 75.0 | 75.0 | 44.0 | 66.9 | 66.7 | 63.1 | **(*f_K_*)^2^** | 74.0 | 61.1 | 68.0 | 60.3 | 61.6 | 74.0 | 0.2 |
| 7.0 | 78.3 | 78.3 | 68.2 | 68.0 | 74.3 | 61.1 | **(*f_K_*)^3^** | 76.2 | 60.6 | 68.0 | 60.0 | 63.3 | 76.2 | 2.4 |
| -3.2 | 68.0 | 67.5 | 51.0 | 68.0 | 65.0 | 54.6 | **asin(*f_K_*)** | 72.0 | 68.3 | 68.0 | 63.6 | 68.3 | 72.0 | -1.9 |
| 0.0 | 77.5 | 77.5 | 62.6 | 67.5 | 64.7 | 60.7 | ***f_N_*** | 75.7 | 40.6 | 68.0 | 65.0 | 59.3 | 75.7 | 0.0 |
| 0.4 | 78.0 | 78.0 | 29.3 | 68.0 | 66.7 | 67.1 | **ln(*f_N_*)** | 73.1 | 61.5 | 68.0 | 67.5 | 66.0 | 73.1 | -2.7 |
| 3.2 | 80.7 | 80.7 | 64.9 | 68.0 | 69.5 | 62.6 | $\frac{\mathbf{1}}{\boldsymbol{f}_{\boldsymbol{N}}}$ | 81.1 | 57.7 | 68.0 | 73.3 | 70.6 | 81.1 | 5.4 |
| -2.1 | 75.4 | 75.4 | 47.2 | 67.5 | 61.6 | 62.1 | $\sqrt{\boldsymbol{f}_{\mathbf{N}}}$ | 73.6 | 56.2 | 68.0 | 64.1 | 65.3 | 73.6 | -2.1 |
| -5.1 | 72.4 | 72.4 | 41.0 | 68.0 | 57.3 | 60.5 | **(*f_N_*)^2^** | 76.6 | 66.9 | 68.0 | 67.5 | 59.6 | 76.6 | 0.9 |
| -4.5 | 73.0 | 73.0 | 67.0 | 68.0 | 65.9 | 63.1 | **(*f_N_*)^3^** | 76.0 | 48.9 | 68.0 | 67.9 | 59.7 | 76.0 | 0.3 |
| 0.5 | 78.0 | 78.0 | 64.2 | 68.0 | 65.5 | 64.0 | **asin(*f_N_*)** | 76.7 | 53.4 | 67.5 | 62.7 | 64.6 | 76.7 | 1.0 |
| 0.0 | 83.0 | 83.0 | 56.4 | 68.0 | 70.9 | 63.4 | ***f_R_*** | 75.1 | 43.0 | 68.0 | 65.5 | 54.7 | 75.1 | 0.0 |
| -5.4 | 77.5 | 77.5 | 54.9 | 68.0 | 66.7 | 55.5 | **ln(*f_R_*)** | 74.8 | 34.9 | 68.0 | 65.8 | 58.9 | 74.8 | -0.3 |
| -2.3 | 80.7 | 80.7 | 51.7 | 68.0 | 69.5 | 52.8 | $\frac{\mathbf{1}}{\boldsymbol{f}_{\boldsymbol{R}}}$ | 72.2 | 68.3 | 68.0 | 67.9 | 60.3 | 72.2 | -2.9 |
| -4.1 | 78.9 | 78.9 | 61.8 | 68.0 | 72.4 | 59.0 | $\sqrt{\boldsymbol{f}_{\mathbf{R}}}$ | 74.7 | 53.2 | 68.0 | 67.9 | 55.4 | 74.7 | -0.4 |
| -2.7 | 80.2 | 80.2 | 67.4 | 68.0 | 70.1 | 63.4 | **(*f_R_*)^2^** | 79.8 | 61.1 | 68.0 | 73.0 | 56.3 | 79.8 | 4.6 |
| 0.0 | 83.0 | 83.0 | 55.6 | 68.0 | 75.0 | 62.4 | **(*f_R_*)^3^** | 76.7 | 51.6 | 68.0 | 69.6 | 66.3 | 76.7 | 1.5 |
| -5.0 | 78.0 | 78.0 | 50.6 | 67.5 | 68.8 | 63.0 | **asin(*f_R_*)** | 78.0 | 50.3 | 68.0 | 64.5 | 54.3 | 78.0 | 2.8 |
| 0.0 | 73.9 | 73.9 | 62.4 | 66.9 | 49.7 | 56.8 | ***f_E_*** | 71.2 | 61.0 | 67.5 | 59.2 | 50.9 | 71.2 | 0.0 |
| -2.9 | 70.9 | 70.9 | 59.6 | 68.0 | 55.5 | 65.1 | **ln(*f_E_*)** | 77.5 | 48.5 | 67.5 | 66.3 | 46.4 | 77.5 | 6.3 |
| -2.9 | 70.9 | 70.9 | 54.2 | 67.5 | 61.8 | 69.4 | $\frac{\mathbf{1}}{\boldsymbol{f}_{\boldsymbol{E}}}$ | 77.8 | 57.3 | 66.4 | 68.9 | 45.4 | 77.8 | 6.6 |
| -3.3 | 70.5 | 70.5 | 57.5 | 68.0 | 52.3 | 58.2 | $\sqrt{\boldsymbol{f}_{\mathbf{E}}}$ | 76.2 | 54.7 | 66.4 | 65.8 | 48.2 | 76.2 | 5.1 |
| -5.8 | 68.0 | 67.8 | 51.0 | 68.0 | 54.1 | 54.8 | **(*f_E_*)^2^** | 76.1 | 51.7 | 67.5 | 64.3 | 57.6 | 76.1 | 5.0 |
| -2.9 | 70.9 | 70.9 | 48.3 | 68.0 | 64.2 | 56.3 | **(*f_E_*)^3^** | 75.1 | 65.5 | 67.5 | 70.2 | 62.0 | 75.1 | 4.0 |
| -3.3 | 70.5 | 70.5 | 57.0 | 68.0 | 57.7 | 65.1 | **asin(*f_E_*)** | 77.1 | 51.2 | 67.5 | 66.7 | 46.4 | 77.1 | 5.9 |
